# Photolysis of caged cytokinin in single cells of *Arabidopsis thaliana*

**DOI:** 10.1186/s13007-022-00953-4

**Published:** 2022-11-11

**Authors:** Lachlan Dow, Russell A. Barrow, Rosemary G. White, Ulrike Mathesius

**Affiliations:** 1 Division of Plant Sciences, Research School of Biology, The Australian National University, Canberra, ACT 2601 Australia; 2 Graham Centre for Agricultural Innovation, Charles Sturt University, Wagga Wagga, NSW 2678 Australia; 3 CSIRO Agriculture and Food, Canberra, ACT 2601 Australia

**Keywords:** Cytokinin, Multiphoton microscopy, Root development, Caged cytokinin, Caged adenine

## Abstract

**Background:**

Cytokinins are a class of phytohormone that play a crucial role in the development of plants. They are involved in the regulation of nearly every aspect of plant growth, from germination to senescence. The role of cytokinins in many developmental programs is complex and varies both spatially and temporally. Current techniques used to investigate the functions of cytokinins in plant development lack this spatial and temporal resolution required to observe cell-type specific effects.

**Results:**

To this end, we present a method of activating a caged cytokinin in single cells. A caged benzyladenine was synthesized, along with caged adenine as a negative control. In vitro testing confirmed ultraviolet light-mediated uncaging, and subsequent root growth assays demonstrated that uncaging produced a cytokinin phenotype. This uncaging was confined to single cells using multiphoton confocal microscopy. Using an *Arabidopsis thaliana* cytokinin reporter line expressing *TCSn::GFP*, the resulting GFP expression was confined to the uncaging region, including in single cells. This study presents a novel cell-targeted method of cytokinin delivery, which has the potential to elucidate a broad range of processes in plant development.

**Conclusions:**

We combined multiphoton confocal microscopy and a caged cytokinin treatment, allowing cell type-specific uncaging of a cytokinin in *Arabidopsis* roots.

**Supplementary Information:**

The online version contains supplementary material available at 10.1186/s13007-022-00953-4.

## Background

Plants respond to environmental inputs through a spectrum of signalling molecules. Cytokinins are evolutionarily old signalling molecules, present in all plant species as well as bacteria, fungi and animals, and the diversity of cytokinin molecules and the roles they play in plant development have increased as land plants have become more complex [1]. The action of cytokinins is mediated by histidine protein kinases (Arabidopsis histidine kinases or AHKs) that act by transferring a phosphoryl group to histidine phosphotransfer proteins. This activates gene transcription through response regulator (Arabidopsis response regulator—ARR)-dependent activation. While some of the ARRs activate transcription of downstream genes (e.g., type-B ARRs), others (e.g., type-A ARRs) negatively regulate cytokinin signalling, including inhibition of type-B ARRs in a negative feedback loop [2]. Many different cytokinins have been identified in plants, although not all of their activities have been demonstrated in vivo.

Cytokinins provide positional signals in plant development, controlling both cell division and differentiation [3]. For example, in the root, cytokinins are involved in the initiation of lateral roots, organisation of the vascular bundle, maintenance of the meristematic zone as well as the transition zone and overall root growth [4]. Similarly, in the shoot, cytokinins control meristem activity, as well as stomatal and flower development. In legume species, cytokinins are also responsible for relaying signals from symbiotic rhizobia in a cell type-specific manner [5].

Cytokinin action is regulated by spatial control of their synthesis and breakdown in different cell types [6], their short and long-distance transport within the plant [7], as well as the responsiveness of different cell types to different cytokinins [8]. For example, localized synthesis of cytokinins induces the division of cortical cells and the formation of nodules in legume roots [9]. Transport of cytokinins from the root to the shoot has been shown to control shoot development in a cytokinin transporter mutant [10]. The individual responsiveness of different cell types to cytokinins has been demonstrated by visualizing cytokinin responses with reporter genes, which demonstrates that not all cells respond equally to external treatment with cytokinins [11].

Knowing the cell type-specific responses to cytokinins is crucial for understanding their role in plant development, with cytokinins often inducing opposing effects in adjacent tissues. For example, while cytokinins inhibit lateral root initiation in the pericycle, they stimulate cortical cell divisions during nodulation. Legume mutants defective in cytokinin perception therefore show a phenotype with increased numbers of lateral roots and decreased numbers of nodules [12]. However, many cell-type specific effects of cytokinins remain unknown, including signalling, sensitivities, and localisation.

Unravelling the action of cytokinins in plant development requires the combination of many approaches. Sensitive techniques for the quantification of cytokinins in tissues have been developed [13], although their detection in situ remains difficult. Cell-specific cytokinin responses have been visualized using reporter constructs such as the *TCSn::GFP* or *LUC* reporters in *Arabidopsis*, which respond specifically to synthetic or natural cytokinins [14]. The *TCSn* promoter is activated by type-B response regulators downstream of the activation of a cytokinin receptor. Such reporters have been used to show that cells differ in their response to cytokinins, and that cytokinin responses in specific cells reflect their biological roles in cell division and differentiation. However, probing the response of different cell types to cytokinins has typically involved treatment of tissues with a cytokinin solution, which does not target individual cell types or single cells.

More specific cytokinin treatments can be achieved through caged probes. Caged probes are molecules that have been rendered biologically inactive through covalent bonding with a chemical ‘cage’, which can then be removed through photolysis, typically with UV light at a certain wavelength [15]. Different caged plant hormones were previously developed [16], including auxin [17] and cytokinins [18]. These caged probes have been used for studies in plant development in particular, as the uncaging can be controlled in space and time to release the bioactive compound. The cage is designed in a way that does not prevent the uptake of the caged molecule into the tissue.

Hayashi and colleagues [18] presented a synthesis for a caged form of benzyladenine, a synthetic form of a cytokinin that is recognized by cytokinin receptors. The authors showed that the caged benzyladenine (CBA) is uncaged with an external UV light from a fluorescence microscope directed at the root tip, and this caused root growth inhibition and induction of the cytokinin responsive *ARR5::GUS* construct in transgenic *Arabidopsis* roots.

Herein we present a novel method with which to probe the cell type-specific effects of cytokinins, by uncaging a cytokinin in single cells using multiphoton confocal microscopy.

## Results

### Synthesis of caged benzyladenine and caged adenine

Prior studies by Hayashi and co-workers [18] successfully developed a number of caged cytokinin molecules, including caged benzyladenine (CBA), and confirmed this molecule inhibited tap root growth following exposure to UV light. We endeavoured to corroborate this study, while exploiting modern microscopy techniques, to confine uncaging in subcellular spaces.

**Fig. 1:**
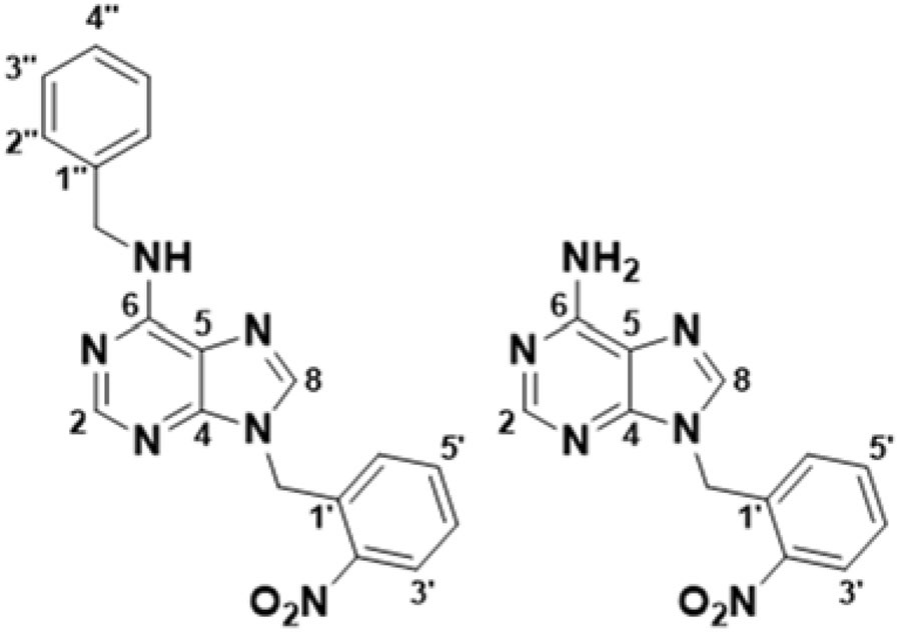
Structures of caged benzyladenine (left), and caged adenine (right), including atom numbering

During the in vitro testing of the uncaging activity of CBA, it became evident that a suitable negative control was required to account for the multiple by-products formed during the uncaging photochemical reaction. These by-products were visible in the UPLC chromatograms produced in quantification experiments (see below). This led to the synthesis of a caged adenine (CA) (Fig. 1), which we hypothesised would be uncaged in a manner similar to caged benzyladenine, while possessing no cytokinin activity. The successful synthesis of CA was achieved by treatment of adenine with either sodium hydride or lithium ethoxide, to produce the respective adenide salts, sodium adenide and lithium adenide in dimethyl formamide. The adenide salt was then treated with 2*-*nitrobenzyl bromide to produce caged adenine that was purified using silica column chromatography. Successful synthesis and purification of both CBA and CA was confirmed using NMR (see Additional file 1: Figs. S1–S10).

### Assessing uncaging efficiency in vitro

Initial uncaging experiments were conducted in vitro, to confirm that photolysis could be achieved with a variety of UV sources. Using the UV source of a Leica M205FA stereo microscope, a solution of CBA was irradiated for up to one hour. The resulting solution was analysed using a UPLC system with photodiode array detector, and this confirmed the photosensitivity of CBA, showing that one of the reaction products matched the retention time and UV absorbance spectrum of benzyladenine (BA) (Additional file 1: Table S1). This experiment was repeated, using a range of UV wavelengths (see Additional file 1: Table S1, S2 and Fig. S11), which showed that CBA is more photosensitive to shorter wavelengths, producing higher concentrations of BA. These tests confirmed the potential to produce active BA with a range of UV wavelengths.

### Caged benzyladenine inhibits root growth after photolysis

**Fig. 2:**
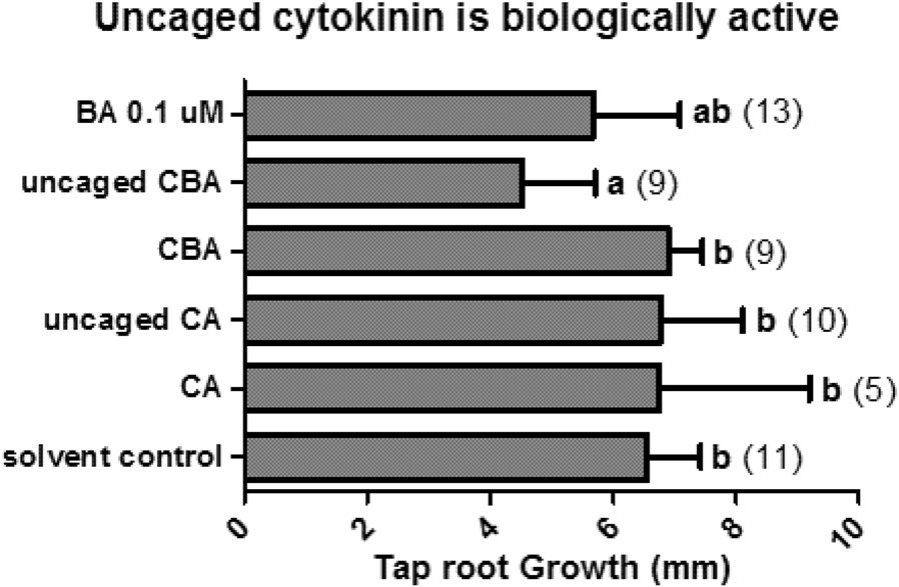
Irradiating a solution of CBA produces a bioactive cytokinin. Two different solutions were uncaged in this experiment: 100 μM CA, and 100 μM CBA. Each solution was irradiated for one hour (see methods), then applied to the root tips of 7-day old *A. thaliana* seedlings, along with the negative controls of CA, CBA (which had not been irradiated) and solvent alone, and the positive control of 0.1 μM BA. The root growth of each cohort was measured after 24 h. Bold letters describe the results of a one-way ANOVA (Dunnett’s post-test, compared to solvent control group, P < 0.05), numbers in brackets correspond to the number of replicates in each treatment, and error bars depict standard deviation

Having confirmed the photosensitivity of CBA, we assessed the bioactivity of CBA post uncaging with two experiments. Firstly, by applying the irradiated (uncaged) CBA to roots, and secondly by applying caged CBA to roots before irradiating the roots themselves. In order to be confirmed as a working caged compound, CBA should inhibit root growth only after the molecule has been exposed to UV light. Thus, there were six treatments in this experiment: negative control (solvent only), positive control (0.1 µM standard BA), UV irradiated CBA solution, non-irradiated CBA solution (UV control), UV irradiated CA (experimental control), non-irradiated CA. Results shown in Fig. 2 demonstrated that UV-irradiated CBA is biologically active, having a significant inhibitory effect on root growth after 24 h (P < 0.05). This effect was stronger than that of 0.1 µM BA, which is plausible if an uncaging efficiency of approx. 11.9% produced an approx. 10 µM solution of free BA from a 100 µM CBA starting solution. Conversely, caged adenine did not alter root growth, thus confirming its viability as a negative control.

**Fig. 3:**
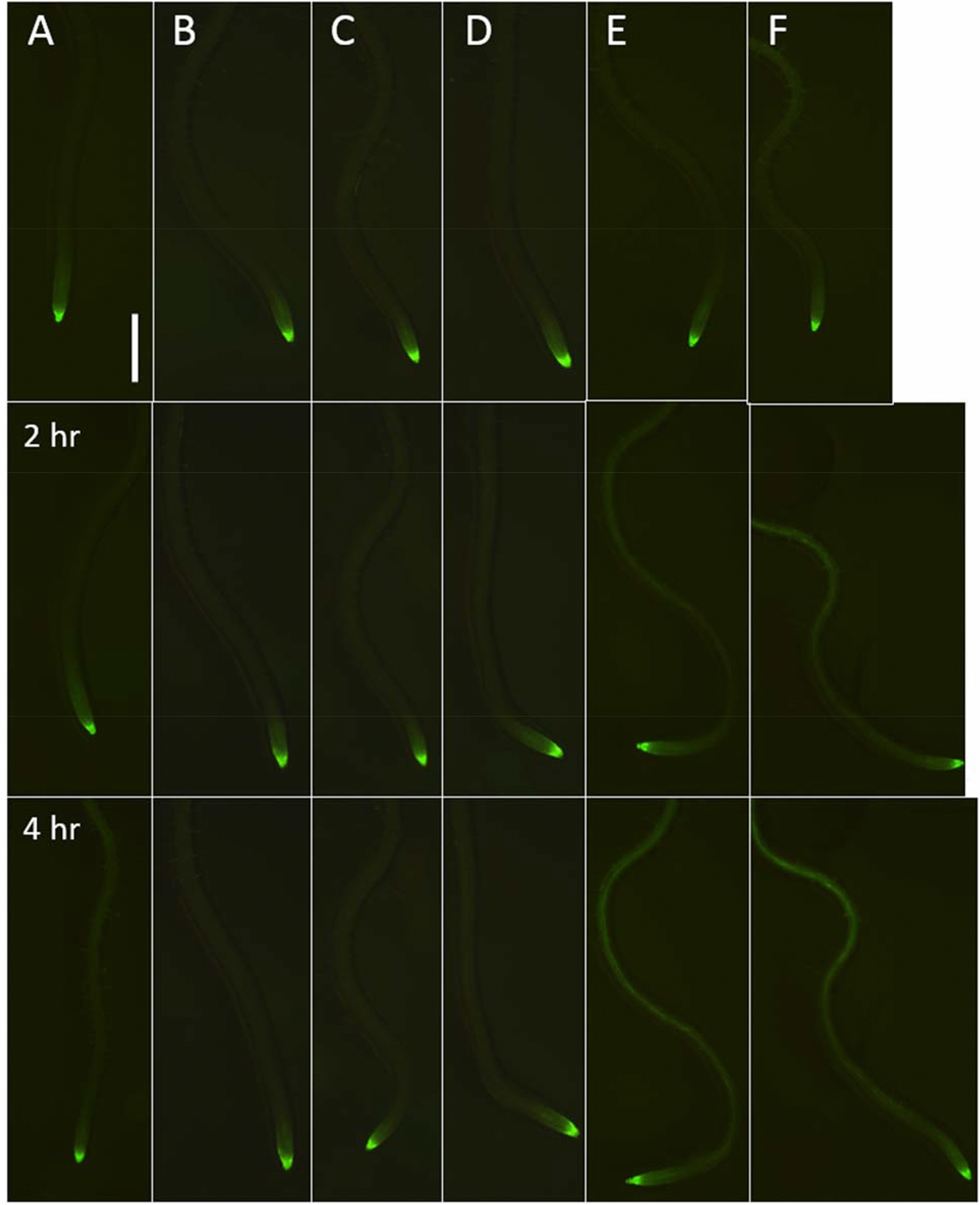
Fluorescence stereomicrographs displaying GFP expression of *TCSn::GFP* roots following uncaging treatments. 7-day old seedlings were treated with CA or CBA, as well as solutions of these molecules that had been previously uncaged by UV illumination for one hour (as well as positive and negative control treatments). The top row shows the roots at time 0. **A** Solvent control (0.5% DMSO); **B** 100 µM CA; **C** 100 µM uncaged CA; **D** 100 µM CBA; **E** 100 µM uncaged CBA; **F** 0.1 µM BA. Scale bar: 500 µm. Quantification of this fluorescence is shown in Additional file 1: Fig. S12

Applying the same solutions to roots of the the *TCSn::GFP* reporter line induced GFP fluorescence only when solutions contained active BA (Fig. 3). Background GFP expression was seen mainly in the root tips at 0 h, similar to published reports for the same reporter [14]. In all roots treated with negative control solutions (0.5% DMSO, non UV-irradiated CBA, non UV-irradiated CA and UV-irradiated CA) this expression pattern was retained over 4 h. In roots treated with UV-irradiated CBA or with 0.1 µM BA, increased GFP fluorescence could be seen along the roots, mainly in the vascular tissue, within 2 h, with a stronger response after 4 h. This result demonstrated that in vitro uncaged CBA produced sufficient free BA to activate the *TCSn* promoter, confirming that short wavelength light is necessary to uncage the CBA. It was also possible to apply CBA directly onto root tips of seedlings and uncage the compound via irradiation of the root tip (Additional file 1: Fig. S12). Uncaging of CBA in vivo reduced root growth in wild type *Arabidopsis* seedlings, although this result was not statistically significant and the inhibition not as severe as with a BA solution, (Additional file 1: Fig. S13) presumably due to the fact that not all CBA was uncaged in situ resulting in a lower BA content.

### Confining photolysis of caged benzyladenine to single cells using multiphoton confocal microscopy

**Fig. 4:**
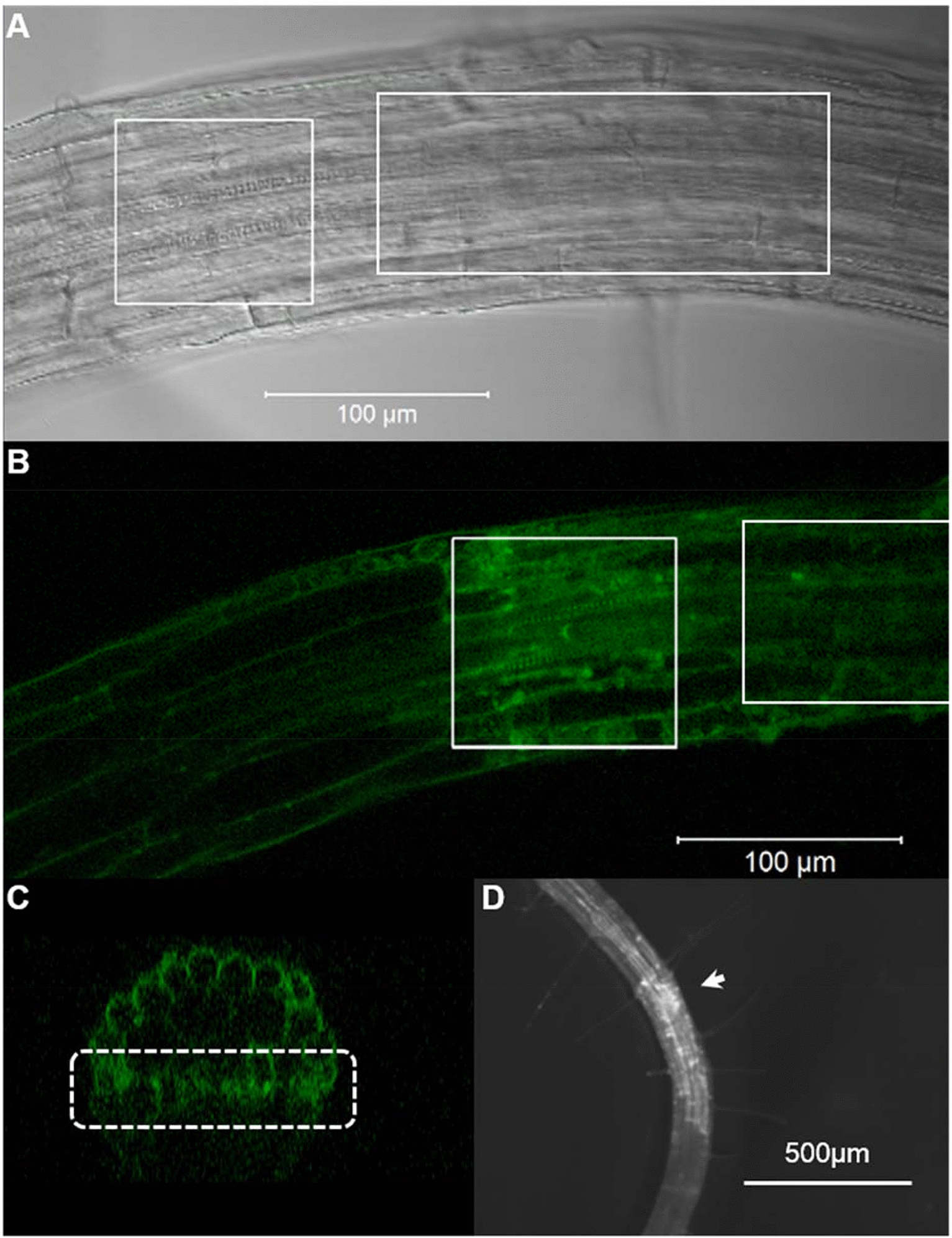
Confocal micrographs displaying multiphoton-induced uncaging of CBA in roots of the *TCSn::GFP* cytokinin reporter line. Roots of 7-day old seedlings were incubated in CBA in the dark for 24 h before irradiation with the 720 nm MP laser. **A** Transmitted light and reflected GFP fluorescence of root tissue before uncaging. **B** fluorescence micrograph of the same root, showing GFP signal approximately two hours post uncaging and confined to the irradiated region. **C** Computational z-section of the same root, produced from a z-stack, demonstrating the vertical specificity of GFP signal, highlighted in dotted white box (as well as background autofluorescence produced in the epidermis). All images captured using 488 nm tuneable argon laser, while uncaging performed using 720 nm MP laser. **D** Strong GFP signal was detected from the same root using fluorescence optics (470 nm) on a Leica M205FA stereomicroscope approximately four hours after irradiation (region indicated by white arrow)

Roots of the *A. thaliana* cytokinin reporter line, *TCSn::GFP* were treated with CBA for 24 h before uncaging with the multiphoton laser of the confocal microscope (see methods). This pulsed laser uses 720 nm light, which is not energetic enough to initiate uncaging reactions. However, at high fluxes at the focal point, infrared photons are dense enough that two may arrive at the caging group simultaneously, acting like a single UV photon and initiating uncaging. Therefore, the goal of this method was to confine the uncaging of benzyladenine to the focal point, while leaving caged cytokinin unaffected above and below the focal point. In initial experiments, large sections of root were irradiated to generate a large signal from the uncaged CBA, as shown in Fig. 4. After approximately two hours, the GFP signal increased and was easily detected using confocal microscopy, and after approximately four hours was also visible using a fluorescence dissecting microscope (Fig. 4D). Illumination of cell walls (see Fig. 7) caused cells to bulge and warp in some cases, suggesting cells were damaged, however these cells were still able to produce a GFP signal in response to uncaged benzyladenine, indicating they were still able to respond to stimuli, transcribe genes and translate mRNA.

**Fig. 5:**
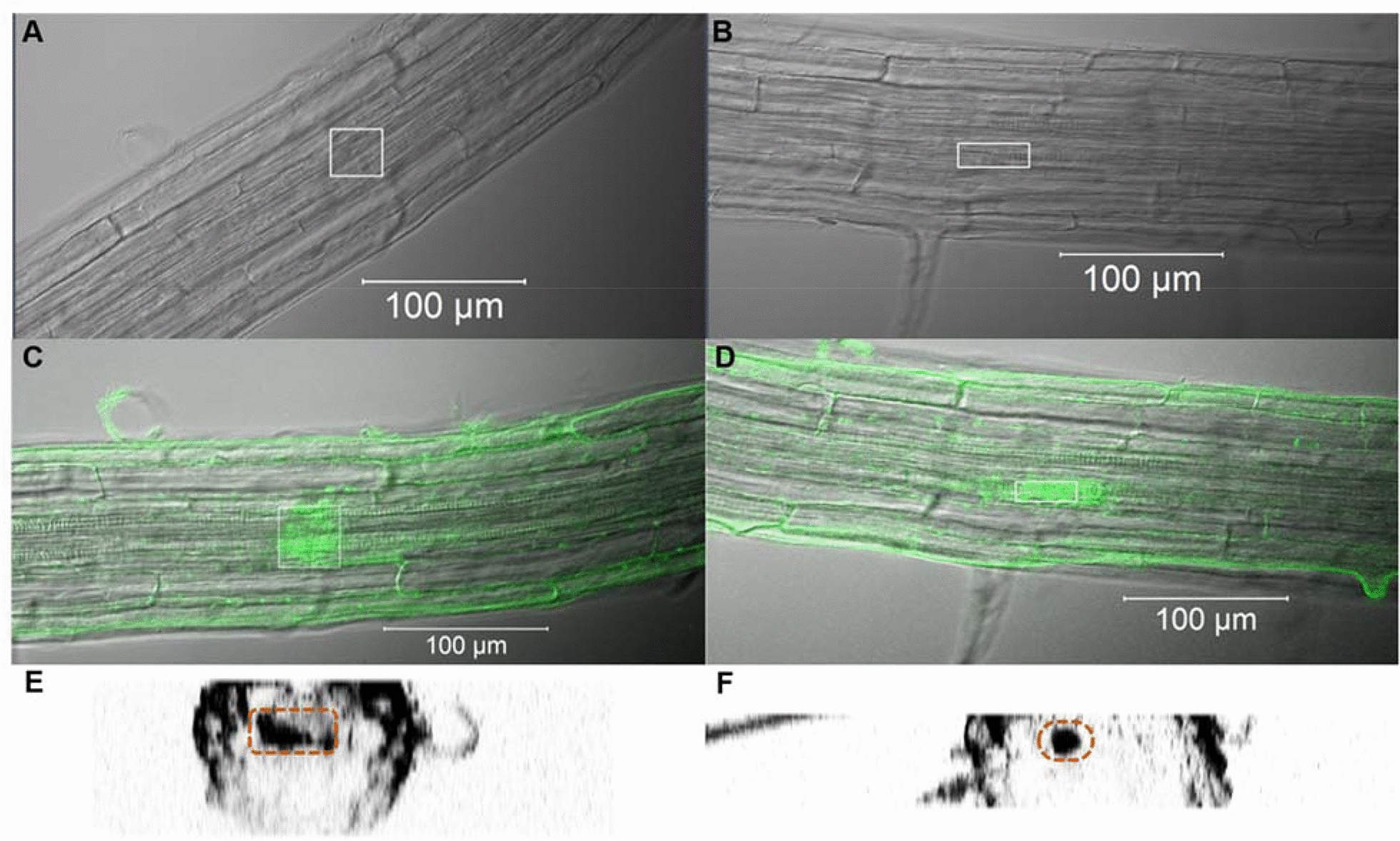
Uncaging CBA in specific regions within root tissues of the cytokinin-responsive *TCSn::GFP* reporter line. **A** and **B** Transmitted light and reflected GFP fluorescence of root tissue before uncaging. **C** and **D** Transmitted light and reflected GFP fluorescence displaying the GFP signal detected after 2 h in the respective irradiated regions (white boxes). **E** and **F** Transverse section from z-stack showing highly localised area expressing GFP (orange dotted lines) (as well as background fluorescence produced in the epidermis). **C** and **E** illustrate cytokinin responses observed in endodermis, pericycle and vascular tissues, while **D** and **F** illustrate cytokinin responses in the endodermis. All images captured using 488 nm tuneable argon laser, while uncaging performed using 720 nm MP laser

More localised tissue targets for CBA uncaging included the central vasculature, cortex, pericycle and epidermal cells, with particular focus on uncaging CBA to generate a bioactive cytokinin in cortical cells. Some cells readily showed GFP fluorescence, especially the vascular tissue, while others showed little or no detectable signal. Where a response was induced successfully, the GFP fluorescence was localised to the region of interest (Fig. 5C and E), and could be confined to single cells (Fig. 5D and F).

**Fig. 6:**
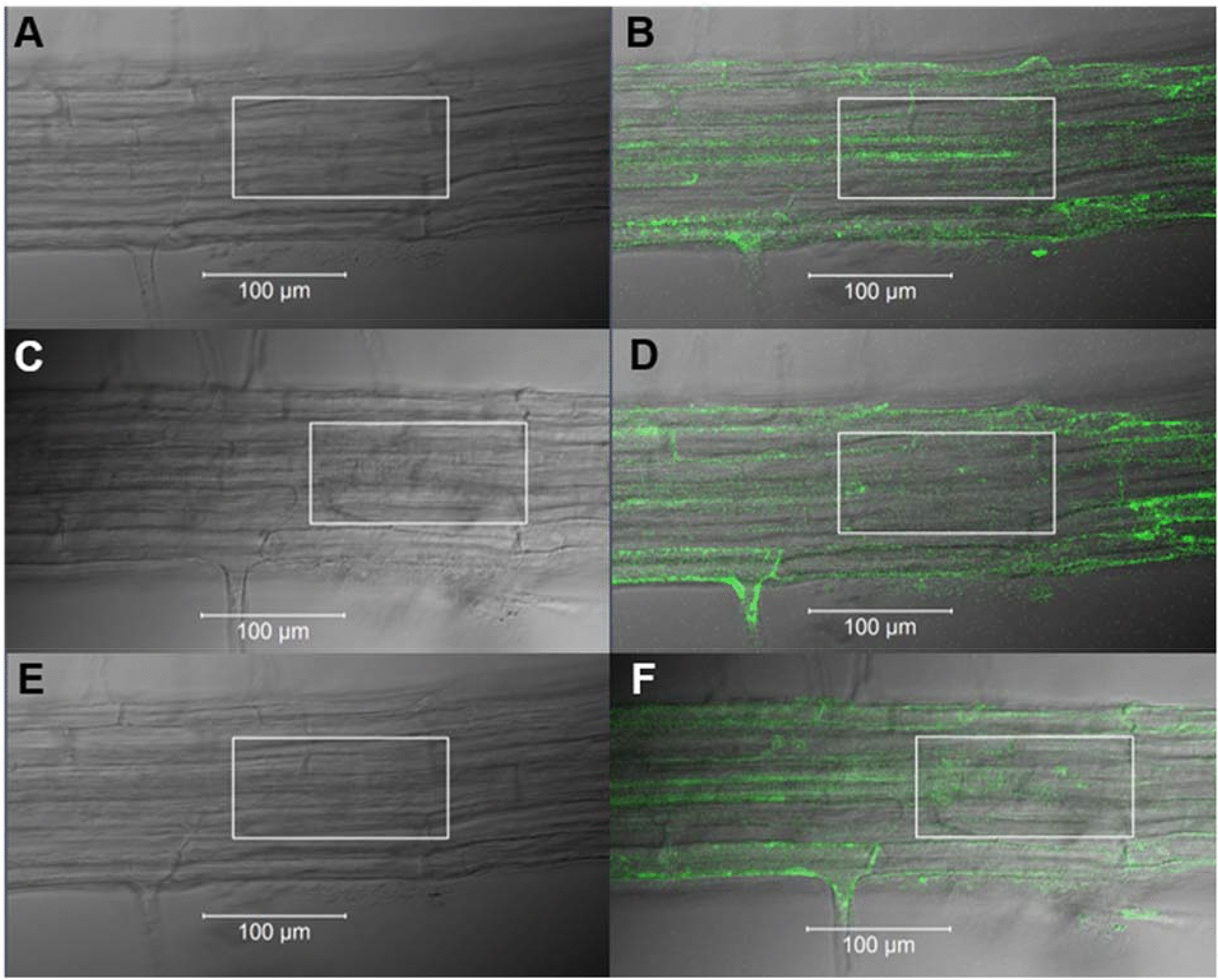
Uncaging caged adenine root tissues of the cytokinin-responsive *TCSn::GFP* reporter line. GFP fluorescence was recorded before (**A** and **B**), immediately after (**C** and **D**), then two hours after (**E** and **F**) irradiation with the 720 nm MP laser (white boxes). All images captured using 488 nm tuneable argon laser, while uncaging performed using 720 nm MP laser. **A**, **C**, **E** Transmitted light images; **B**, **D**, **F** reflected GFP fluorescence images

One concern during analyses of induced fluorescent signals was the contribution of background GFP- or autofluorescence unrelated to the experimental treatment. To address this question, roots were incubated in CA and the standard uncaging protocol was applied to large regions of root tissue. Very little fluorescence was induced in roots of the cytokinin-responsive *TCSn::GFP* reporter line (Fig. 6), showing that the GFP fluorescence observed after uncaging CBA was indeed a response due to the cytokinin BA. Immediately after laser irradiation, root tissue fluorescence decreased, then after two hours returned to a level similar to that prior to irradiation. As noted in Figs. 4, 5, 6, in roots incubated in CBA, this is the time at which induced GFP expression was detected.

**Fig. 7:**
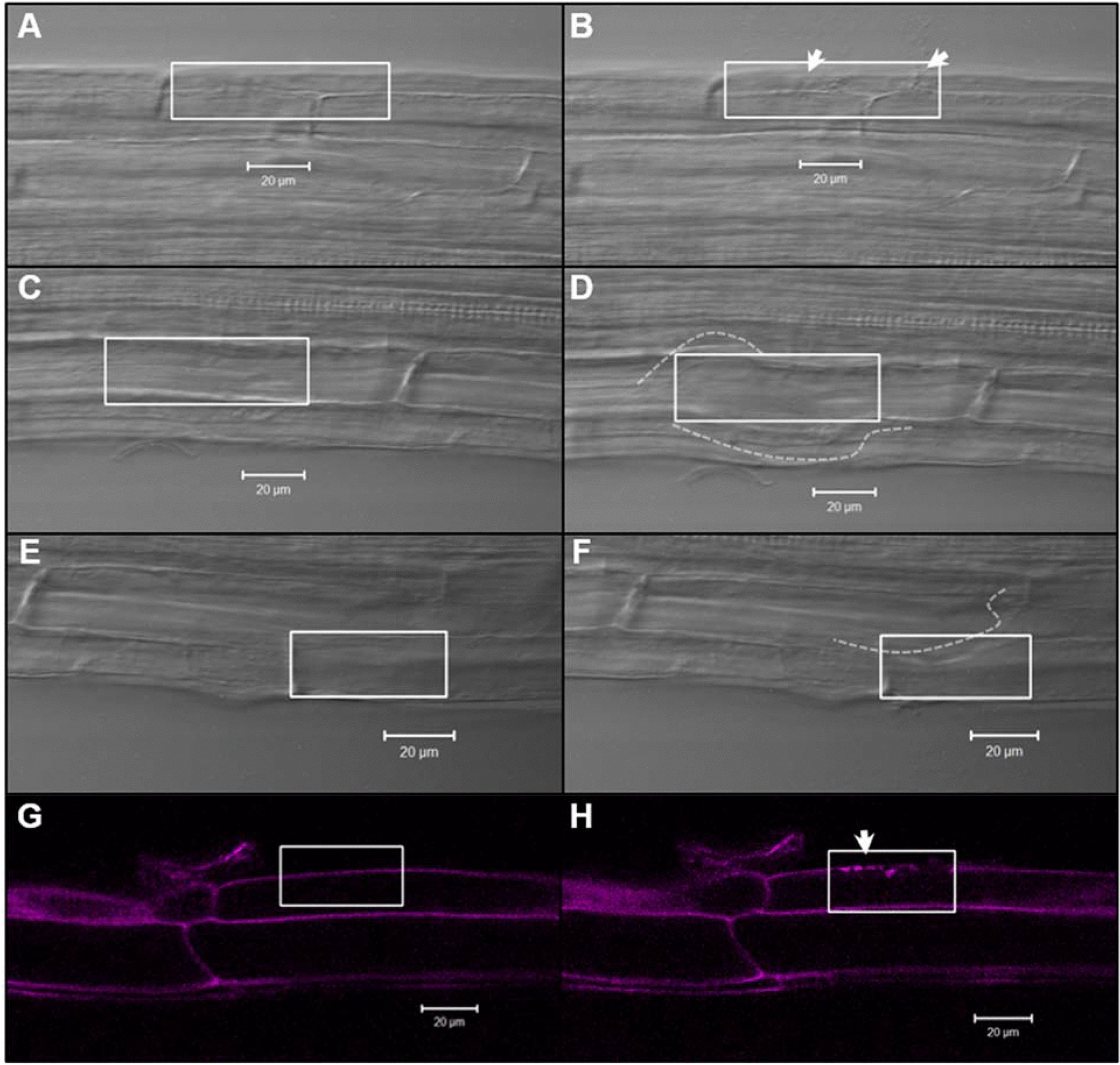
Monitoring cell wall damage following irradiation with a 720 nm MP confocal laser (70% intensity). Cells of *A. thaliana* roots before (**A**, **C**, **E**, **G**), and immediately after (**B**, **D**, **F**, **H**) MP irradiation. White arrows/dotted lines highlight bulges or punctures in the cell walls. Red or yellow boxes indicate region of irradiation. **G** and **H** Propidium iodide (3 mg/ml), applied to the roots ten minutes before irradiation, highlighting cell wall structures, shown in magenta. Images **A**–**F** taken using transmitted light; images **G** and **H** captured using 488 nm reflected fluorescence optics

Cells were monitored for any local damage caused by the uncaging process using the same microscope settings as were used to uncage CBA. By including cell walls within the irradiated region (boxes in Fig. 7), structural damage was more readily visible. Epidermal cell walls were most susceptible while others were left unchanged, as seen in earlier studies [19]. Walls were also stained with propidium iodide (3 mg/ml for ten minutes) to highlight any damage (Fig. 7 G, H; 488 nm excitation, 500-540 emission). Given that most cells (when cell walls were not targeted) were unscathed by irradiation, and the clear ability of irradiated *TCSn::GFP* cells to transcribe and translate GFP (see Figs. 4 and 5), we concluded that the cellular machinery of plant roots remained functional during 720 nm MP uncaging.

Uncaging experiments were attempted, focussing on a range of tissue types and caged molecules. These results are summarised in Table 1, highlighting the reliability and accuracy of the method, along with the cell-type specific sensitivity to cytokinin. For example, experiments using CA induced no GFP response, as expected from a molecule with no cytokinin activity. Various tissue types were irradiated using CBA, all of which were responsive to CBA uncaging, with the exception of the cortex.

**Table 1:** Overview of uncaging experiments and their success rates

Cell/tissue type targeted	Uncaging experiments attempted	Fluorescence responses observed in targeted area	Example image
Caged fluorescein	11	10	Additional file 1: Fig. S16
CA	3	0	Figure 6
CBA, Large region (multiple tissues)	5	5	Figure 4
CBA, Vascular bundle	3	3	Figure 5C
CBA, Pericycle/endodermis	1	1	Figure 5D
CBA, Cortex	23	0	None
CBA, Epidermis	1	1	Additional file 1: Fig. S17

## Discussion

This study describes novel methodology to ‘uncage’ a caged cytokinin in single cells. This was achieved through the synthesis of CBA and the negative control caged adenine. The biological activity of uncaged CBA was confirmed, through root growth assays, and it was determined that this was due to uncaging CBA to produce BA. Importantly, CBA was only biologically active after exposure to UV light. Uncaging of CBA inhibited root growth, and promoted GFP expression in the *Arabidopsis* cytokinin reporter line *TCSn::GFP*. This was achieved with a range of different UV sources such as microscopes equipped with fluorescence optics (Fig. 3, Additional file 1: Fig. S12), and confocal microscopes equipped with infrared multiphoton optics (Figs. 4 and 5). Subsequent experiments demonstrated that CBA can be uncaged in a cell-specific manner. This was achieved by uncaging CBA with a multiphoton confocal microscope. Finally, CBA was successfully uncaged in single cells using this technique and the cellular response observed via GFP expression in the *TCSn::GFP* line. The uncaging efficiency of this method was not measured, however the clear GFP expression in the reporter line confirmed that biologically relevant concentrations were comfortably reached. A caged auxin was previously uncaged in single *N. tabacum* suspension cells [17], however to our knowledge this is the first time a caged phytohormone has been uncaged in specific cell types and tissues of a plant.

Interestingly, no GFP signal could be observed when single cortical cells were targeted for CBA uncaging (see Table1), as observed using cytokinins in previous studies [11]. While *Arabidopsis* cortical cells are not thought to be sensitive to cytokinins, it should be noted that cortical cells are relatively large, and most of their volume is taken up by the vacuole. If any CBA within the vacuole were to be uncaged, it would not have activated any cytokinin receptors given AHK is not expected to localise to the vacuole plasma membrane [20].

There is considerable chemical diversity in naturally produced cytokinins [21], and the individual roles played by these cytokinins (and corresponding glycosides) is not clear. While BA is a synthetic cytokinin, Hayashi and colleagues also synthesised a caged dihydrozeatin, a naturally occurring cytokinin, highlighting the opportunity for more types of cytokinins to be synthesised in the future [18]. With the addition of multiphoton confocal microscopy techniques described here, the authors hope that outstanding biological questions regarding the role of cytokinins in plant development can be elucidated.

## Conclusions

We developed a method, including negative controls, for the uncaging of the phytohormone cytokinin (the synthetic cytokinin benzyl adenine) in defined regions or single cells of *Arabidopsis*. This method could be useful for testing cell type-specific cytokinin responses in various plant species, although the cumulative effect of increasingly thicker tissues on multiphoton optics might pose a limitation in larger plants. In *Arabidopsis*, all cell types, including the vascular tissue in the root, could be targeted with laser powers that kept cells alive and able to respond with altered gene expression. While the *TCSn:GFP* reporter line was useful to observe cytokinin responses, the method described here is also usable in plant species without this reporter gene.

## Methods

### Synthesis of caged benzyladenine

Where used, percentage solution concentrations are reported as v/v and were made in distilled water unless stated otherwise. The synthesis of caged benzyladenine (CBA) was based on the synthesis devised by Hayashi et al. [18] with minor modifications (specifically, butanol was not used as a solvent during the substitution of chlorine with benzyl amine). To a suspension of 6-chloropurine (694 mg, 4.49 mmol) in tetrahydrofuran (6 mL), 2*-*nitrobenzyl bromide (2041 mg, 9.44 mmol) and 1,8- diazabicycloundec-7-ene (1075 mg, 69.4 mmol) were added, and the reaction mixture stirred for 22 h at room temperature*.* The resulting thick yellow suspension was neutralised with ammonium chloride solution and extracted with ethyl acetate. The ethyl acetate solution was washed with saturated sodium chloride solution and the solvent removed under reduced pressure, yielding a yellow solid (1.727 g). This crude material was dissolved in neat benzylamine (1065 mg, 9.9 mmol) and the reaction mixture stirred at room temperature for 5 h. The reaction was neutralised with ammonium chloride solution, extracted with ethyl acetate, washed with saturated aqueous sodium chloride, dried with anhydrous MgSO_4_ and evaporated under reduced pressure, yielding crude CBA (*N*-benzyl-9-(2-nitrobenzyl)-9*H*-purin-6-amine) (374 mg). The crude solid was purified by trituration by placing on filter paper and washing repeatedly with ethyl acetate to return the desired compound. Synthesis and purification were confirmed using NMR and mass spectrometry (see Additional file 1: Fig. S1–S5 for spectra and NMR assignment).

### Synthesis of caged adenine

Following the methodology described in the publications of Rasmussen, Beasley and Hope, [22–24], a suspension of adenine, (139 mg, 1.03 mmol) in dry dimethyl formamide (DMF) (50 ml), was degassed and kept under an argon atmosphere. To this reaction vessel, a suspension of sodium hydride in mineral oil (60%, 42 mg, 1.05 mmol) in dry DMF (40 ml) was added via syringe. The reaction mixture was left to stir overnight, after which all of the adenine was dissolved, indicating formation of the sodium adenide salt. A solution of 2*-*nitrobenzyl bromide (221 mg, 1.02 mmol) in dry DMF (10 ml) was then added, and the reaction mixture left to stir at room temperature for 48 h. The reaction was monitored with thin layer chromatography (using ethyl acetate as a mobile phase). The DMF was evaporated at 40 °C under high vacuum conditions and the residue treated with aqueous sodium carbonate (30 ml). The resulting mixture was extracted with multiple extractions with chloroform (6 × 20 ml). The chloroform extracts were combined, dried with anhydrous NaSO_4_, and evaporated to dryness to give a pale yellow solid. Column chromatography on a silica column, eluting with 9:1 dichloromethane:methanol* returned the desired 2-nitrobenzyladenine as a pale yellow solid.

(*treating the methanol with gaseous ammonia immediately before use to produce an alkaline mobile phase significantly improves the solubility of the caged adenine and enhances the chromatography).

### Purification of caged adenine

Gaseous ammonia was bubbled through methanol for approximately five minutes, dissolving the ammonia to produce an ‘ammonium methoxide’ methanol solution. This methanol was then used as the eluent in silica flash column chromatography in a DCM:MeOH solution in 9:1 ratio. Fractions were collected and TLC used to identify the presence of product under UV light. These fractions were then evaporated under vacuum (44% approximate yield). Purification was confirmed using NMR and mass spectrometry (see Additional file 1: Fig. S6–S10 for spectra and NMR assignment).

### Growth conditions

Seeds of *Arabidopsis thaliana* (Col-0 and a transgenic line expressing *TCSn::GFP;* [14]) were surface sterilised, first in 70% ethanol for two minutes, then for ten minutes in 10% sodium hypochlorite and 5 µl Tween. The hypochlorite solution was then removed, and the seeds rinsed five times with sterile water and plated on square Petri dishes (12 cm length) containing half-strength Murashige and Skoog (½ MS) agar media, eight seedlings per plate. Seedlings were incubated in a Thermoline incubator at 21 °C with a 15/9 h day/night cycle at approximately 150 µE light intensity. For all experiments, seedlings were grown for seven days before start of the treatment.

The lights in the incubator were covered with UV400 Covershields (CoverShield, Lancershire, UK), which filtered out wavelengths below 400 nm. This was done to prevent uncaging of CBA or CA in the growth chamber.

#### Assessing uncaging efficiencies in vitro

A volume of 20 µl of 100 µM CBA in 0.5% DMSO was added to the wells of a clear plastic 96 well microtitre plate and irradiated with UV light (340—380 nm) using a Leica M205FA stereomicroscope, approximately 10 cm from the objective lens. The microscope was focussed such that the incident UV radiation was confined to the area of one microtitre well. The wells were exposed for either 0, 1, 10 or 60 min in triplicate. The resulting solutions were then diluted with 40 µl of ultrapure water to ensure an adequate volume was available for UPLC injection.

The amount of uncaged BA produced in solution was then quantified using UPLC on a Dionex UltiMateTM 3000 UPLC coupled with a fluorescence FLD-3400 detector (Thermo Fisher Scientific), by comparing the corresponding absorbance integral at 272 nm with that of a BA standard curve. The following gradient was used: 0–5 min, 5–50% AcN (acetonitrile); 5–6 min, 50–100% AcN; 6–6.5 min, 100% AcN; 6.5–7 min, 100–5% AcN, at a flow rate of 0.2 ml min^−1^. The column was a Kinetex C18 column, dimensions 100 × 2.1 mm, particle size 1.7 µm (Phenomenex). Both AcN and water contained 0.1% acetic acid.

#### In vitro photolysis of CBA to inhibit root growth

A volume of 50 µl of either 100 µM CBA or 100 µM CA in 0.5% DMSO was added to the wells of a 96 well microtitre plate and irradiated as described above, for one hour. The solution was then applied to the root tips of *A. thaliana* seedlings (20 µl per root tip). Negative controls of 20 µl 0.5% DMSO, 20 µl non UV-irradiated CBA and 20 µl non UV-irradiated CA (both 100 µM in 0.5% DMSO) and a positive control of 0.1 µM BA (10 µl, in 0.5% DMSO) were included. Root growth was measured using ImageJ software over 24 h [25]. The same solutions were also applied to seedlings of the *TCSn::GFP* reporter line of *A. thaliana* [14], to test for cytokinin-induced reporter expression (see below). The same solutions were also analysed by UPLC for their active concentrations of free BA and adenine.

### In vitro photolysis of CBA to test *TCSn:GFP* expression in whole *Arabidopsis* seedlings

A solution of 100 µM uncaged CBA derived from the uncaging in a microtitre plate (as described above), along with positive and negative control solutions (solvent control, 0.5% DMSO; 100 µM CA; 100 µM uncaged CA; 100 µM CBA; and 0.1 µM BA) was applied to whole *Arabidopsis* seedlings encoding the *TCSn:GFP* reporter. Seedlings were kept on agar plates containing ½ MS medium for the 4 h assay period. A volume of 200 µl solution for each compound was applied along each root. Roots were photographed under a Leica M205FA stereo microscope after excitation with an ET Blue LP filter system (excitation 470 nm, emission 515 nm long pass filter) at the start of the treatment, at 2 h and at 4 h after treatment. All photos were taken with a Leica DFC550 digital camera using identical settings. Plants were kept in the light in the growth chamber (as described above) for the treatment time (except when photographed).

### Cell targeted uncaging growth conditions

**Fig. 8:**
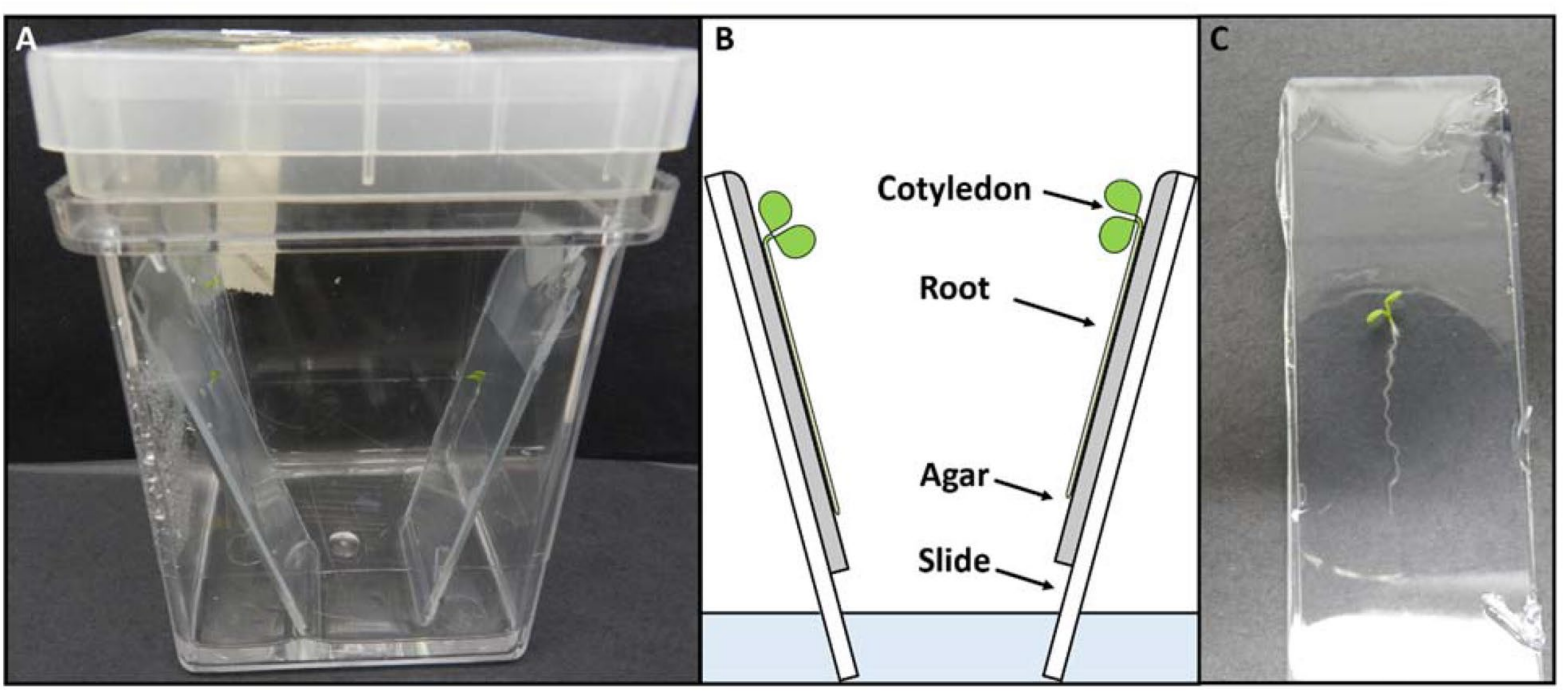
Experimental setup for growing *A. thaliana* seedlings for confocal microscopy. **A** *A. thaliana* seedlings grown inside sterile ‘Magenta’ jars **B** Schematic of seedlings that have germinated and grown on ½  MS agar (grey), on microscope slides (clear). The slides are embedded in ½ MS agar in the base of a Magenta jar. **C** *A. thaliana* seedling on an agar slide prior to removal of the top and bottom ends of the agar medium

Sterilised *A. thaliana* seedlings were grown on a thin layer (approximately 3 mm thick) of ½ S agarose media, on top of a sterile (autoclaved) glass microscope slide. These were housed in a sterile Magenta jar (an air-tight plastic jar designed for tissue culture), which contained a layer of½  MS agarose media (approximately 1 cm deep). A 2 cm strip of agarose was removed from one end of each slide, and the slide embedded in the Magenta jar agarose medium. The uncoated side of the slide rested on the wall of the jar, and a 5 mm strip of agarose was removed from the other (top) end of the slide so that this agarose medium was not disturbed while loading the slides onto the microscope stage. This arrangement served to keep the slides held firmly in place, while also keeping the atmosphere of the jars moist. Sterilised seeds were sown on the slide, approximately 1 cm from the upper end of the remaining growth media. Up to three seedlings could be grown on a single slide, and up to four slides placed in a single Magenta jar. The apparatus is shown in Fig. 8. Seedlings were incubated in a Thermoline incubator at 21 °C with a 15/9 h day/night cycle at approximately 150 µE light intensity. As with previous experiments, the lights in the incubator were covered with UV400 Covershields to filter out wavelengths below 400 nm.

### Confocal microscopy settings

All confocal images were collected on a Zeiss LSM 780 NLO-UV multiphoton confocal laser scanning microscope. The objective used was a W Plan-Apochromat 20x/1.0 DIC M27 water immersion lens, which required no coverslip. Fluorescence from GFP or uncaged fluorescein was obtained using an excitation wavelength of 488 nm, produced from a tuneable argon laser, and emission waveband of 500–540 nm. Fluorescence images were overlaid on a transmitted light channel. Micrographs were processed using the confocal Zen software (Black edition, Zeiss).

### Calibration of multiphoton irradiation

A single *A. thaliana* root was treated with 5 µl of caged fluorescein solution (1 mg/ml, Sigma). After ten minutes, regions of this root were irradiated using a tuneable Mai Tai EHP Deepsee (Spectra Physics) multiphoton laser, set to 720 nm. At sufficient intensity (typically at the focal point of the objective), at least two photons of this wavelength will interact with a single chromophore simultaneously, producing the equivalent effect of approximately 360 nm excitation light. The roots were irradiated using different laser powers, from 40 to 100% of total laser power (which at 720 nm is 1790 mW) in ten percent increments, and the fluorescence response observed (see Additional file 1: Fig. S14). This experiment allowed the determination of the minimum multiphoton laser power required to uncage caged fluorescein effectively and served as a benchmark for further experimentation using the novel caged compounds.

### Multiphoton uncaging of CBA

Seedlings of *A. thaliana* cytokinin reporter line *TCSn::GFP*, which develops visible GFP fluorescence roughly two hours after application of BA, were grown on slides as described above. Roots were treated with 10 µl of CBA solution (100 µM CBA in 0.5% DMSO) and left for 24 h in the dark to ensure no CBA was uncaged by incident light. While the CBA solution did eventually run down the nearly vertical slide, a comparatively large amount of solution wetted the root. The solution was applied to the roots in situ, to keep the roots at the same growing angle, and to limit any potential contamination that could be introduced by moving them.

For each experiment, before uncaging CBA-treated roots, a test root was incubated in caged fluorescein as described above, to ensure correct operation of the confocal system. Roots treated with CBA were then imaged using a tuneable argon laser set at 488 nm, and bleached using a tuneable Mai Tai EHP Deepsee (Spectra Physics) multiphoton laser, set at a wavelength of 720 nm and laser intensity of 70%. Pixel size and pixel dwell time were held at 0.25 µm and 1.9 µs, respectively, throughout all uncaging experiments. These parameters also ensured that each chromophore was irradiated at least once on average. Based on the theoretical diffraction limited spot size for a multiphoton confocal microscope, with the objective used and for 720 nm light, lateral resolution is 0.24 µm. Thus, the diffraction limited spot produced via bleaching is no smaller than the scanning resolution, meaning no tissue was left unbleached between each linear scan of the 720 nm laser. The transmitted light image was optimised using Dodt gradient contrast [26]. Approximately two hours after uncaging CBA, the roots were re-examined (with 488 nm excitation laser with a filter setting of 493–602 nm, along with the transmitted light channel) to detect cytokinin-induced GFP fluorescence. During long intervals between images (which could be several hours), the slides were returned to their Magenta jars to keep roots from drying.

## Supplementary Information

**Additional file 1: Figure S1.** Structure of caged benzyladenine, showing atom numbering system. **Figure S2.** ^1^H NMR spectrum of Caged benzyladenine in CDCl_3_. **Figure S3.** ^13^C NMR spectrum of caged benzyladenine in CDCl_3_. **Figure S4.** Low resolution (**A**) and high resolution (**B**) ESI mass spectra of caged benzyladenine. **Figure S5.** Infrared spectrum of caged benzyladenine, as powder. **Figure S6.** Structure of caged adenine, showing atom numbering system. **Figure S7.** ^1^H NMR spectrum of caged adenine, in d_6_-DMSO. **Figure S8.** ^13^C NMR of caged adenine, in d_6_-DMSO. **Figure S9.** Low resolution (**A**) and high resolution (**B**) ESI mass spectra of caged adenine. **Figure S10.** IR spectrum of caged adenine, as powder. **Table S1:** Assessment of CBA uncaging efficiencies in vitro. **Figure S11.** UPLC chromatograms of benzyladenines. **Table S2.** Effect of UV wavelength on uncaging efficiency. **Figure S12.** Emission spectra of the three different UV lights utilised in S 13. **Figure S13.** ImageJ analysis of fluorescence from images shown in Fig. 3**. Figure S14.** Uncaging in small regions using a compound microscope. **Figure S15.** Uncaging CBA in vivo inhibits root growth. **Figure S16.** Detection of caged-fluorescein uncaging using a 720 nm (multiphoton) laser. **Figure S17.** Further images of successful GFP response from tissue-specific uncaging of CBA.

## Data Availability

All data generated or analysed during this study are included in this published article and its additional information files.

## Abbreviations

BA: Benzyladenine
CA: Caged adenine
CBA: Caged benzyladenine
NMR: Nuclear magnetic resonance
