# Supplementary material for: Photolysis of caged cytokinin in single cells of Arabidopsis thaliana
Source: Plant Methods. 2022 Nov 11;18:120. doi: 10.1186/s13007-022-00953-4 (PMC9652950; doi:10.1186/s13007-022-00953-4)
Supplement: Supplementary file 1 — Additional file 1: Figure S1. Structure of caged benzyladenine, showing atom numbering system. Figure S2. 1H NMR spectrum of Caged benzyladenine in CDCl3. Figure S3. 13C NMR spectrum of caged benzyladenine in CDCl3. Figure S4. Low resolution (A) and high resolution (B) ESI mass spectra of caged benzyladenine. Figure S5. Infrared spectrum of caged benzyladenine, as powder. Figure S6. Structure of caged adenine, showing atom numbering system. Figure S7. 1H NMR spectrum of caged adenine, in d6-DMSO. Figure S8. 13C NMR of caged adenine, in d6-DMSO. Figure S9. Low resolution (A) and high resolution (B) ESI mass spectra of caged adenine. Figure S10. IR spectrum of caged adenine, as powder. Table S1: Assessment of CBA uncaging efficiencies in vitro. Figure S11. UPLC chromatograms of benzyladenines. Table S2. Effect of UV wavelength on uncaging efficiency. Figure S12. Emission spectra of the three different UV lights utilised in S 13. Figure S13. ImageJ analysis of fluorescence from images shown in Fig. 3. Figure S14. Uncaging in small regions using a compound microscope. Figure S15. Uncaging CBA in vivo inhibits root growth. Figure S16. Detection of caged-fluorescein uncaging using a 720 nm (multiphoton) laser. Figure S17. Further images of successful GFP response from tissue-specific uncaging of CBA. [file 13007_2022_953_MOESM1_ESM.docx]

**Addition al file 1**

*Spectral acquisition parameters*

NMR spectra were recorded at 298 K, at 400 MHz for ^1^H, 100 MHz for ^13^C, on a Bruker Ascend 400 instrument. Chemical shifts are reported in ppm (δ). NMR experiments were run in deuterated chloroform (CDCl_3_) or deuterated dimethylsulfoxide (d_6_-DMSO) as indicated. ^1^H NMR spectra are referenced to the resonance from residual CHCl_3_ at 7.26 ppm and CD_3_S(O)CHD_2_ at 2.50 ppm respectively, ^13^C NMR spectra are referenced to the central peak in the signals from CDCl_3_ at 77.0 ppm or d_6_-DMSO at 39,5 ppm.

The multiplicities of ^1^H and ^13^C NMR spectra are expressed by the abbreviations: br (broad), s (singlet), d (doublet), t (triplet), m (multiplet). ^13^C NMR spectra and spectra were run as proton decoupled experiments. ^1^H and ^13^C signals where appropriate are described by chemical shift δ (multiplicity, integration, *J* (Hz), assignment). NMR assignments are supported by 2D NMR spectra (data not shown).

IR spectra were recorded on Bruker Alpha FTIR with diamond plate Attenuated Total Reflectance sampling attachment, run at 4 cm^-1^ resolution. Peak intensities are described by s (strong), m (medium), w (weak), br (broad).

Electrospray ionisation mass spectrometry (ESI-MS) and high resolution (HRESI-MS) were recorded on a Bruker Apex 3. Mass spectra are displayed as mass/charge ratios (*m/z*) and relative abundance (% of base peak intensity).

### *Caged benzyladenine properties, including ^1^H NMR* & *^13^C NMR assignment*

*N*-benzyl-9-(2-nitrobenzyl)-9*H*-purin-6-amine. Melting point: 193 °C. R_f_: 0.4 (EtOAc). UV-VIS: λ_max_: 267 nm (logε 4.23), 211.5 nm (logε 4.30). IR (cm^-1^): 3276 (br), 1624 (s), 1520 (s), 1345 (s). ^1^H NMR (CDCl_3_): 8.42 (1H, s, C2-**H**), 8.17 (1H, d, J=8.26 Hz, C6’-**H**), 7.89 (1H, s, C8-**H**), 7.57 (1H, t, J=8.3 Hz, C5’-**H**), 7.50 (1H, t, J=7.8 Hz, C4’-**H**), 7.41 (2H, m, C3’’-**H**), 7.35 (2H, m, C2’’-**H**), 7.30 (1H, m, C4’’-**H**), 7.14 (1H, d, J=7.76 Hz, C3’-**H**), 6.10 (1H, s (br), C6-N**H**), 5.80 (2H, s, C1’-C**H_2_**), 4.89 (2H, s (br), C1’’-C**H_2_**). ^13^C NMR (CDCl_3_): 154.9 (**C**6), 153.8 (**C**2), 147.8 (**C**4), 147.7 (**C**2’), 140.3 (**C**8), 138.5 (**C**1’’), 134.4 (**C**5’), 131.7 (**C**3’), 130.0 (**C**4’), 129.4 (**C**2’’), 128.9 (**C**3’’), 128.0 (**C**4’’), 127.7 (**C**4’), 125.6 (**C**6’), 121.1 (**C**5), 44.5 (C1’-**C**H_2_), 44.5 (C1’’-**C**H_2_). NB - the triplets observed in the nitrobenzyl moiety in these compounds is considered to be a doublet of doublets, as expected by the two A_1_M_1_X_1_ systems, which has not been resolved with this NMR instrument.

**Figure S1**: Structure of caged benzyladenine, showing atom numbering system.


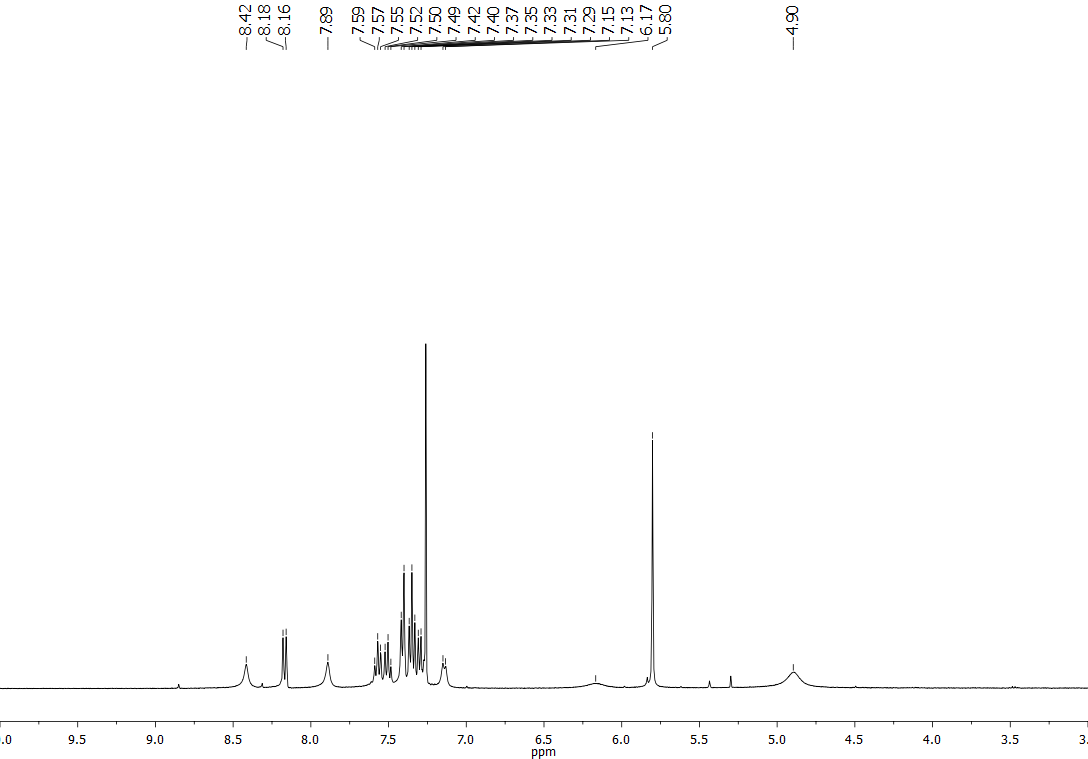


**Figure S2**: ^1^H NMR spectrum of Caged benzyladenine in CDCl_3_.


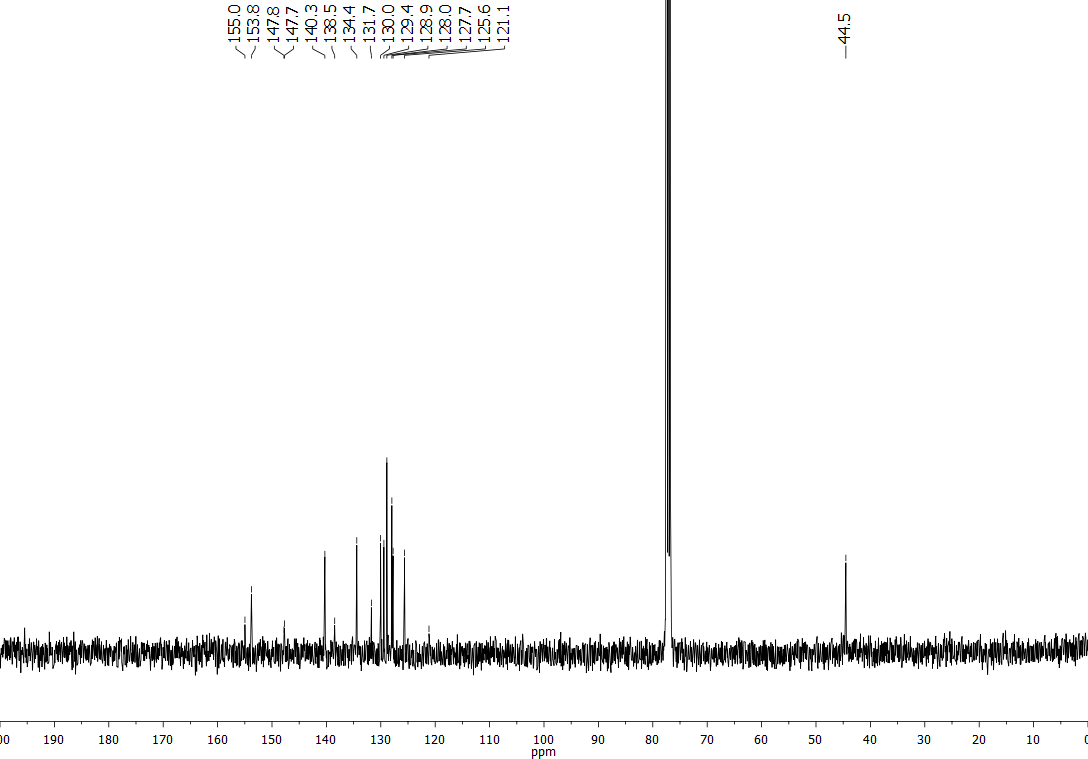


**Figure S3**: ^13^C NMR spectrum of caged benzyladenine in CDCl_3_.


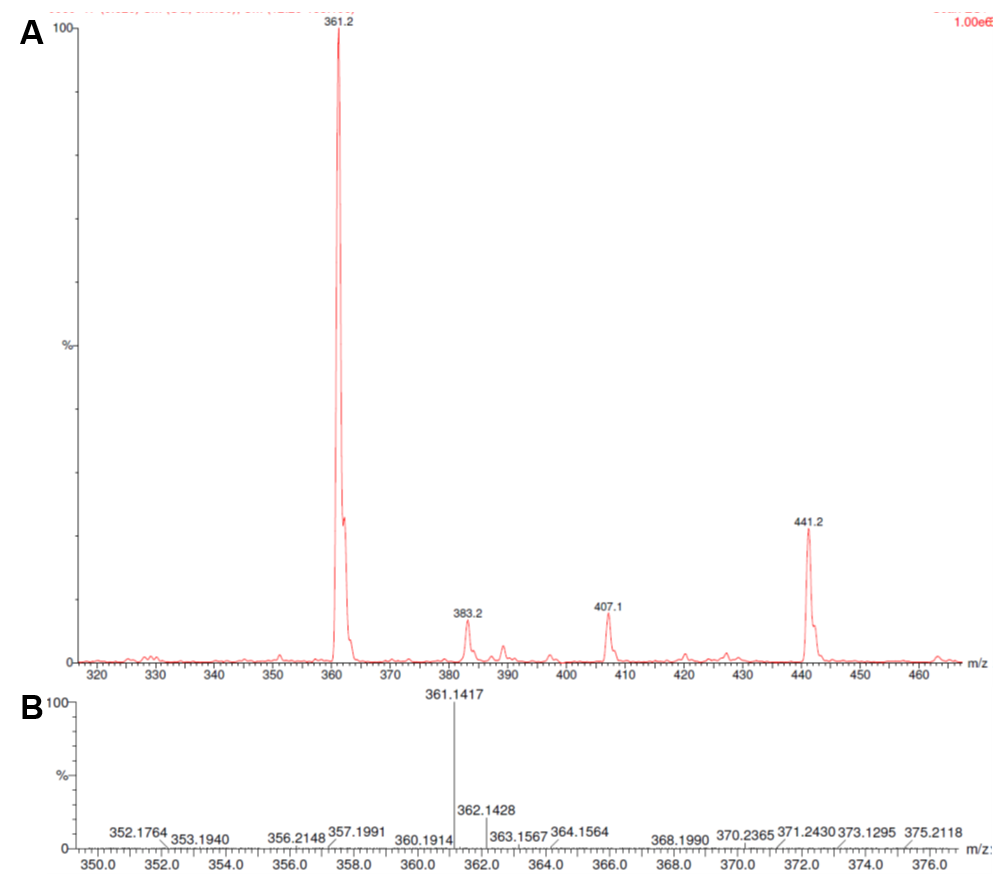


**Figure S4**: Low resolution (**A**) and high resolution (**B**) ESI mass spectra of caged benzyladenine.


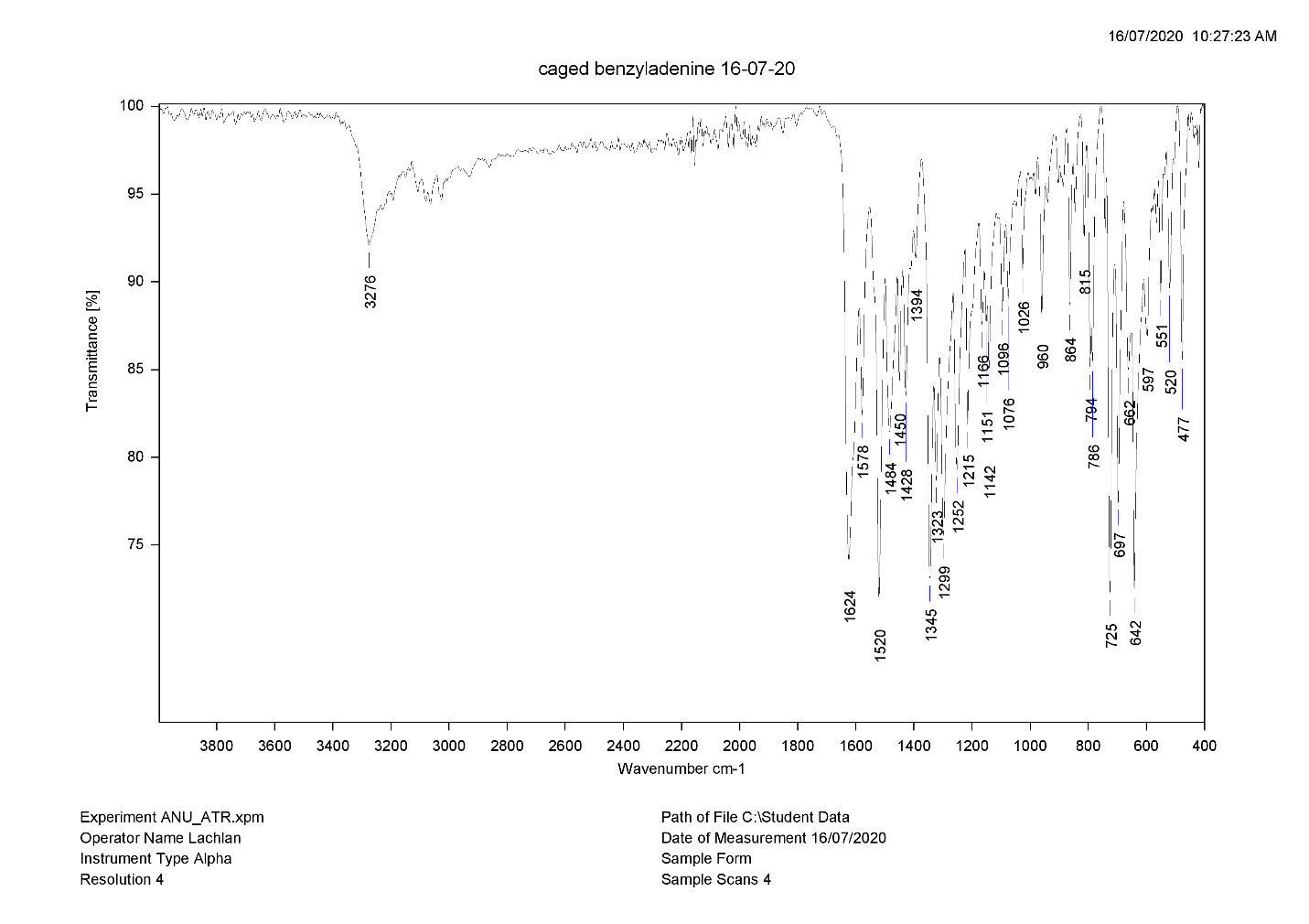


**Figure S5**: Infrared spectrum of caged benzyladenine, as powder.

**Figure S6**: Structure of caged adenine, showing atom numbering system

### Properties of caged adenine, including ^1^H NMR and ^13^C NMR assignment

9-(2-nitrobenzyl)-9*H*-purin-6-amine. Melting point 272-275 °C. R_f_: 0.3 (DCM:MeOH 9:1) UV-VIS: λ_max_: 259.5 nm (logε 3.62), 221.5 nm (logε 3.73). IR (cm^-1^): 3081 (w, br), 1520 (s), 1340 (s), 1300 (s), 641 (m). ^1^H NMR (d_6_-DMSO): 8.18 (1H, s, C2-**H**), 8.15 (1H, d J=7.8 Hz, C6’-**H**), 8.10 (1H, s, C8-**H**), 7.67 (1H, t, J=7.6Hz, C5’-**H**), 7.58 (1H, t, J=7.7Hz, C4’-**H**), 7.31 (2H, s (br), C6-N**H_2_**), 6.90 (1H, d, J=7.7Hz, C3’-**H**), 5.73 (2H, s, C1’-C**H_2_**). ^13^C NMR (DMSO): 156.0 (**C**6), 152.8 (**C**2), 151.3 (**C**2’), 147.3 (**C**1’), 141.0 (**C**8), 134.3 (**C**5’), 132.0 (**C**4), 129.0 (**C**4’), 128.8 (**C**3’), 125.0 (**C**6’), 114.0 (**C**5), 43.4 (C1’**C**H_2_). NB - the triplets observed in the nitrobenzyl moiety in these compounds is considered to be a doublet of doublets, as expected by the two A_1_M_1_X_1_ systems, which has not been resolved with this NMR instrument.
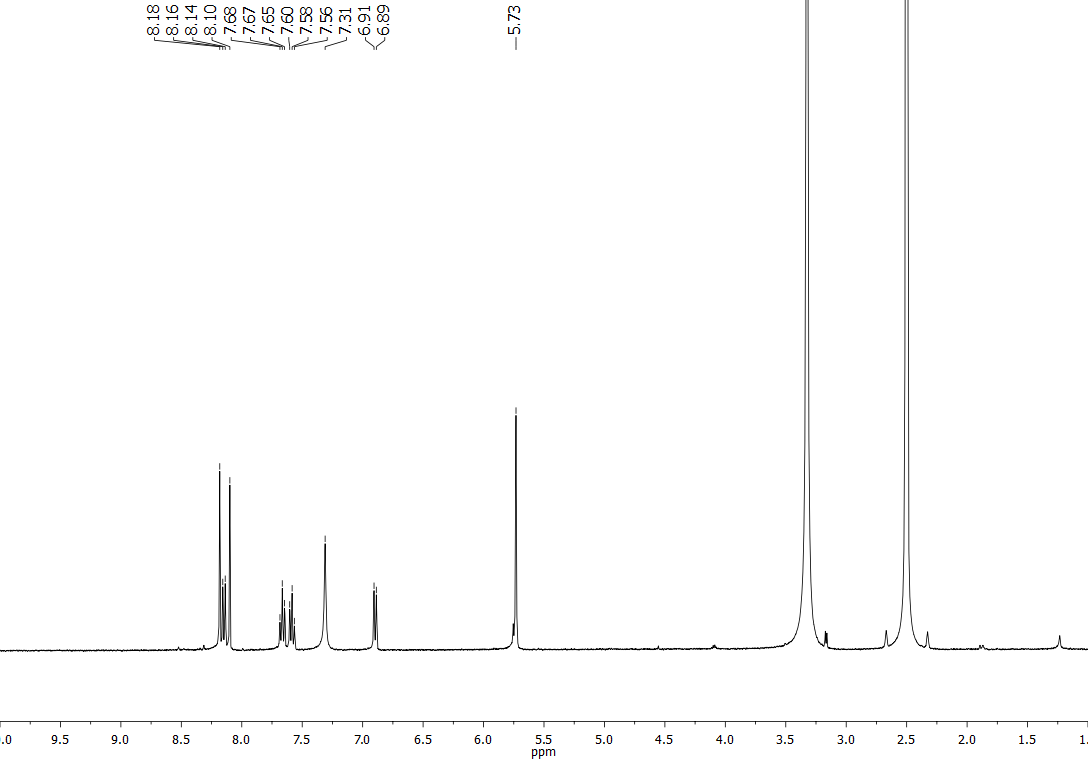


**Figure S7**: ^1^H NMR spectrum of caged adenine, in d_6_-DMSO.


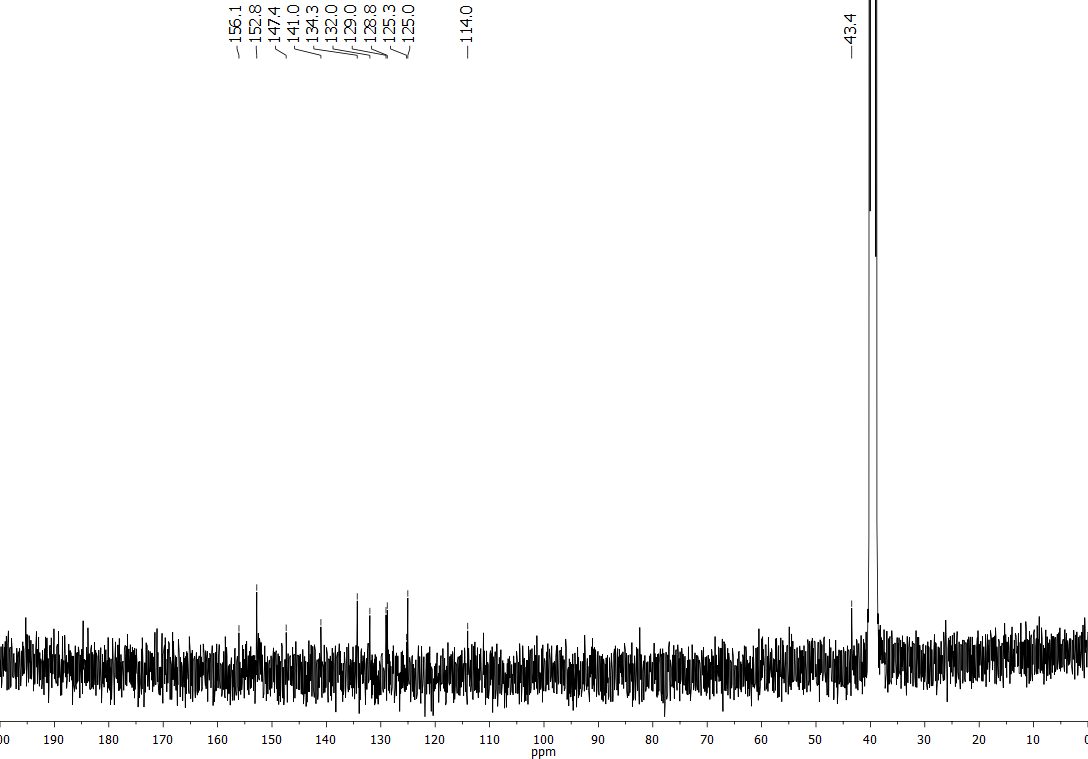


**Figure S8**: ^13^C NMR of caged adenine, in d_6_-DMSO.


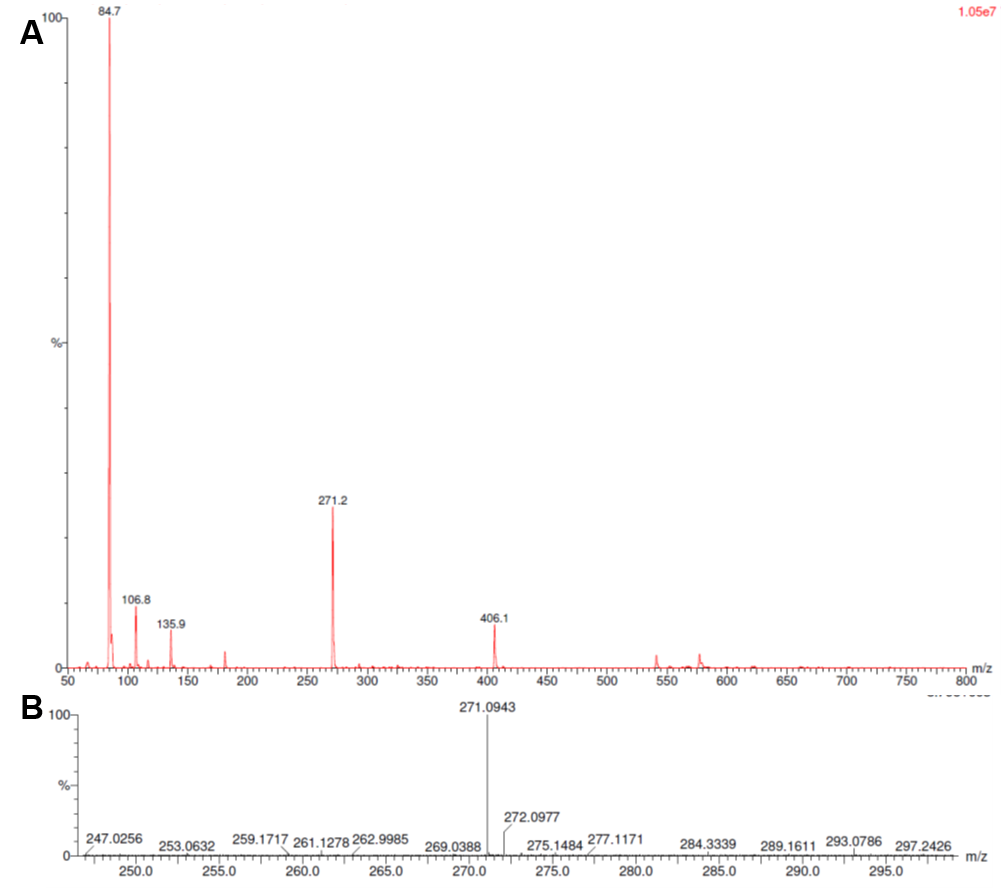


**Figure S9**: Low resolution (**A**) and high resolution (**B**) ESI mass spectra of caged adenine.


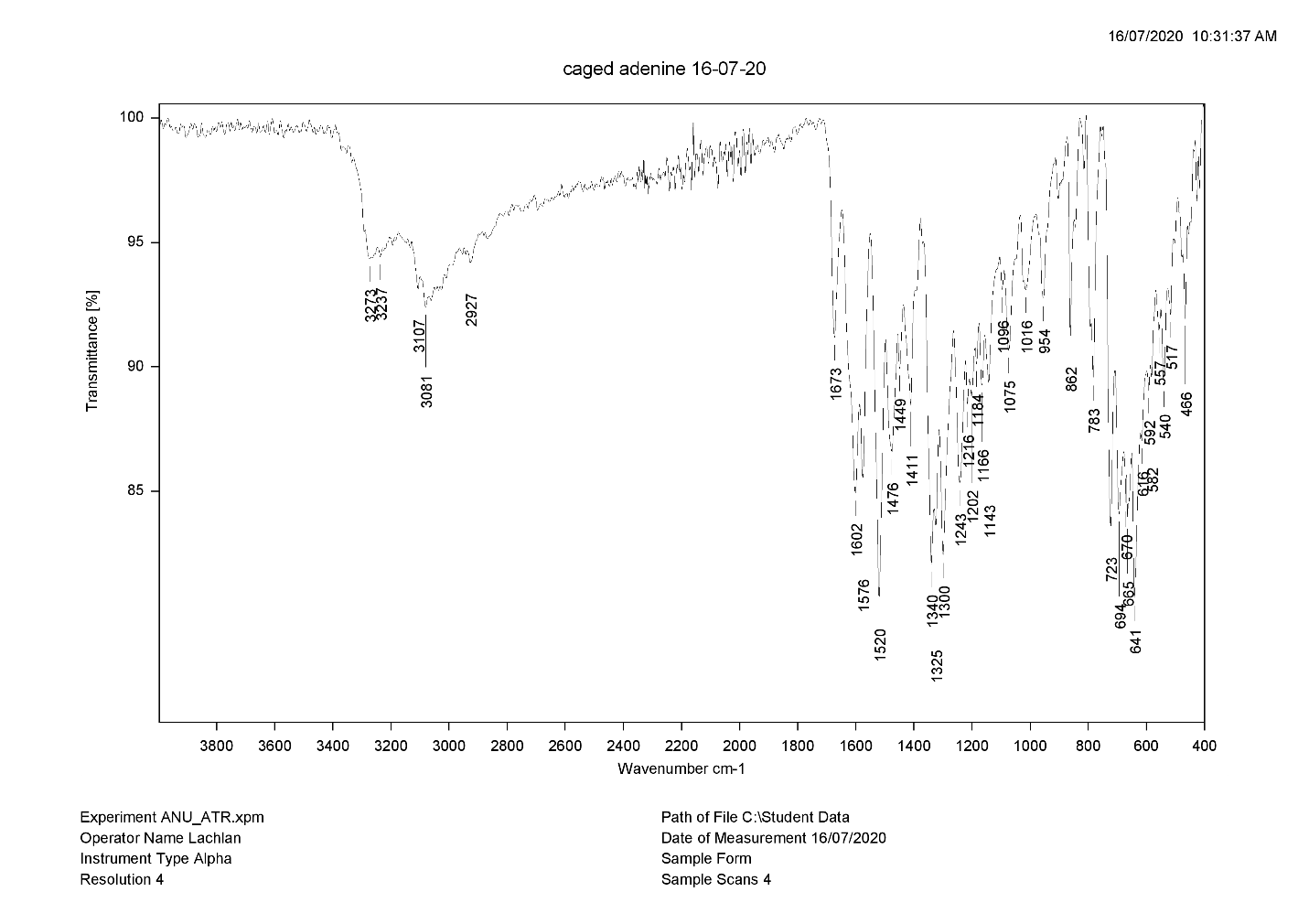


**Figure S10**: IR spectrum of caged adenine, as powder.

**Table S1: Assessment of CBA uncaging efficiencies *in vitro***

| UV wavelength (nm) | Irradiation time (min) | Uncaging efficiency^1^ (%) | ±StDev (n=3) |
| --- | --- | --- | --- |
| 340-380 | 0 | 0 | 0 |
| 340-380 | 1 | 0 | 0 |
| 340-380 | 10 | 8.6 | 2.46 |
| 340-380 | 60 | 11.9 | 5.22 |

^1^ defined as the molar ratio of uncaged BA produced to the CBA starting concentration


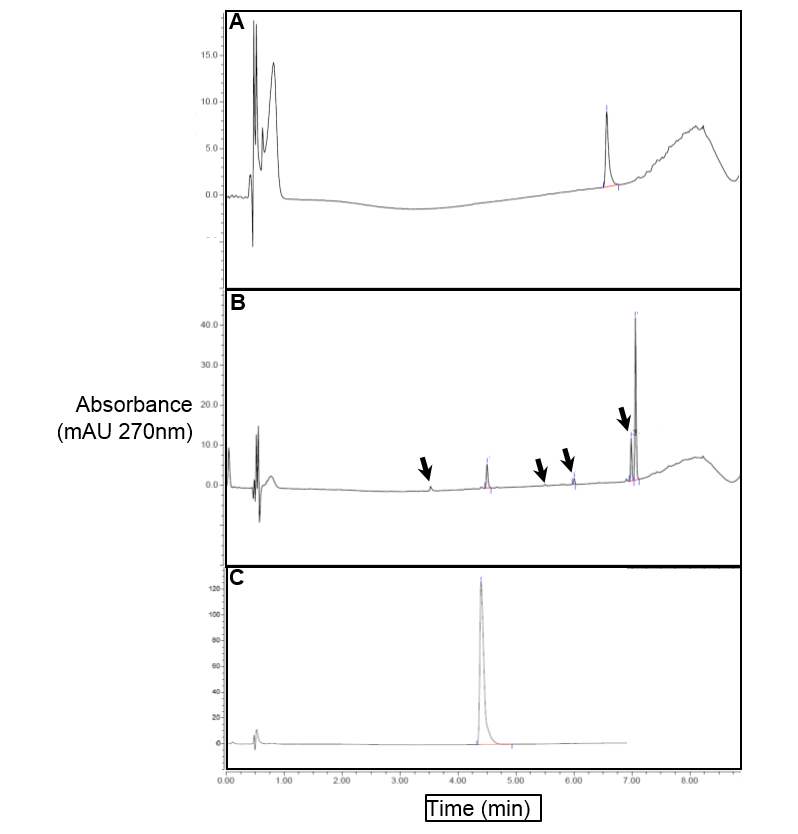


**Figure S11**: UPLC chromatograms of benzyladenines. **A**) 100 μM CBA before uncaging, showing a single peak at approximately 6.5 min retention time. **B**) 100 μM CBA after one hour UV irradiation showing multiple peaks including one eluting at 4.5 min. **C**) 100 μM BA standard eluting at 4.5 min. Based on the multiple peaks observed in **B** (arrows), a negative control, caged adenine, was devised to account for the production of these by-products of uncaging.

**Table S2**: Effect of UV wavelength on uncaging efficiency

| UV wavelength (nm) | Irradiation time (min) | Uncaging efficiency^1^ (%), n=1 |
| --- | --- | --- |
| 315-400 | 180 | 4.54 |
| 280-315 | 180 | 12.5 |
| mostly 254 | 180 | 15.0 |

1: defined as the molar ratio of uncaged BA produced to the starting CBA starting concentration

Using a UV light apparatus, three solutions of 20 µM CBA (100 µl) were irradiated with one of three different UV lights. These lights corresponded to UV-A light, UV-B or UV-C bandpass light, (SI 13 **A, B** and **C** respectively). The solutions were placed 30 cm underneath two UV fluorescent lights (Luzchem Canada), and irradiated for 3 hours, and the resulting BA concentrations quantified via the UPLC methods outlined earlier.


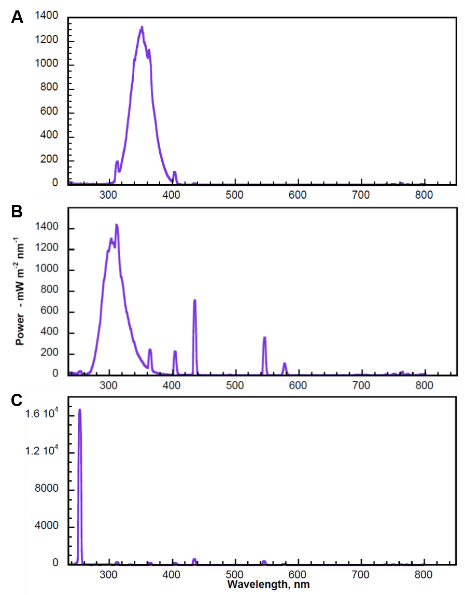


**Figure S12**: Emission spectra of the three different UV lights utilised in SI 12. Note the scale is different in each chart.


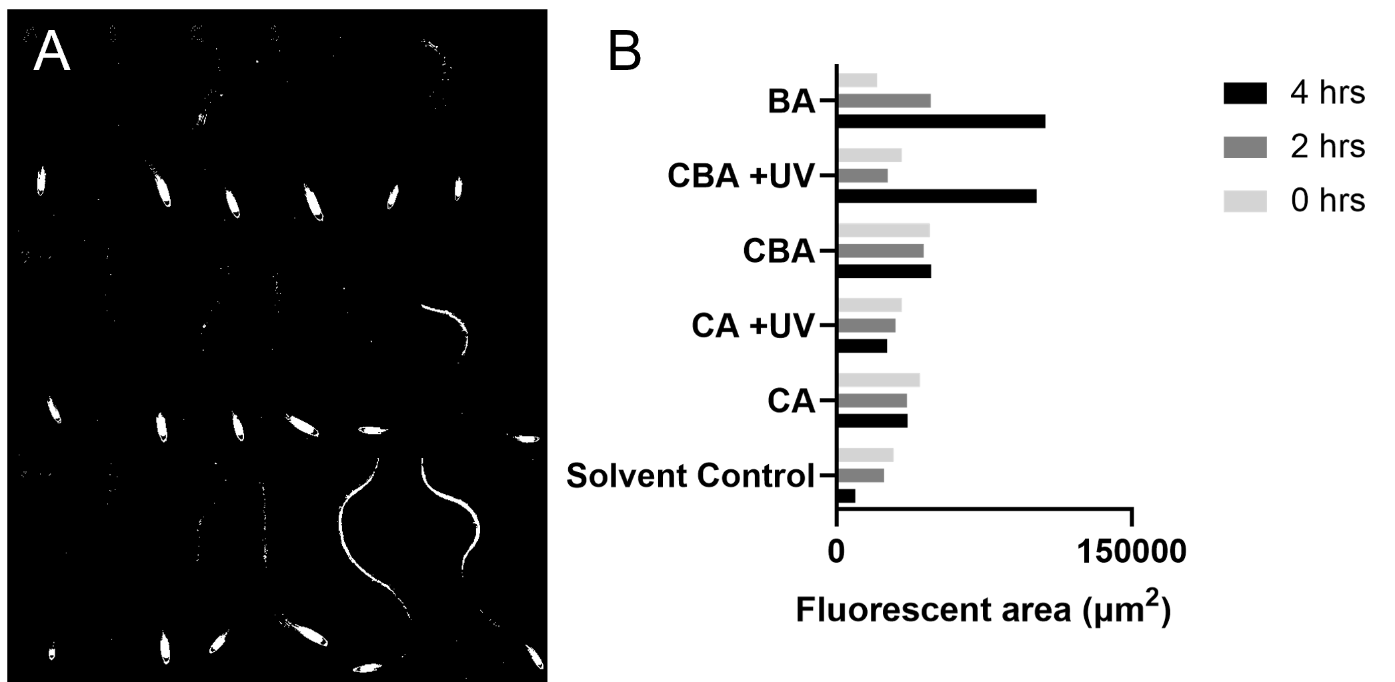


**Figure S13**: ImageJ analysis of fluorescence from images shown in **Figure 3**. The fluorescent area above a certain threshold is shown in panel A. This area is quantified for each root at the respective time points in the graph (panel B).


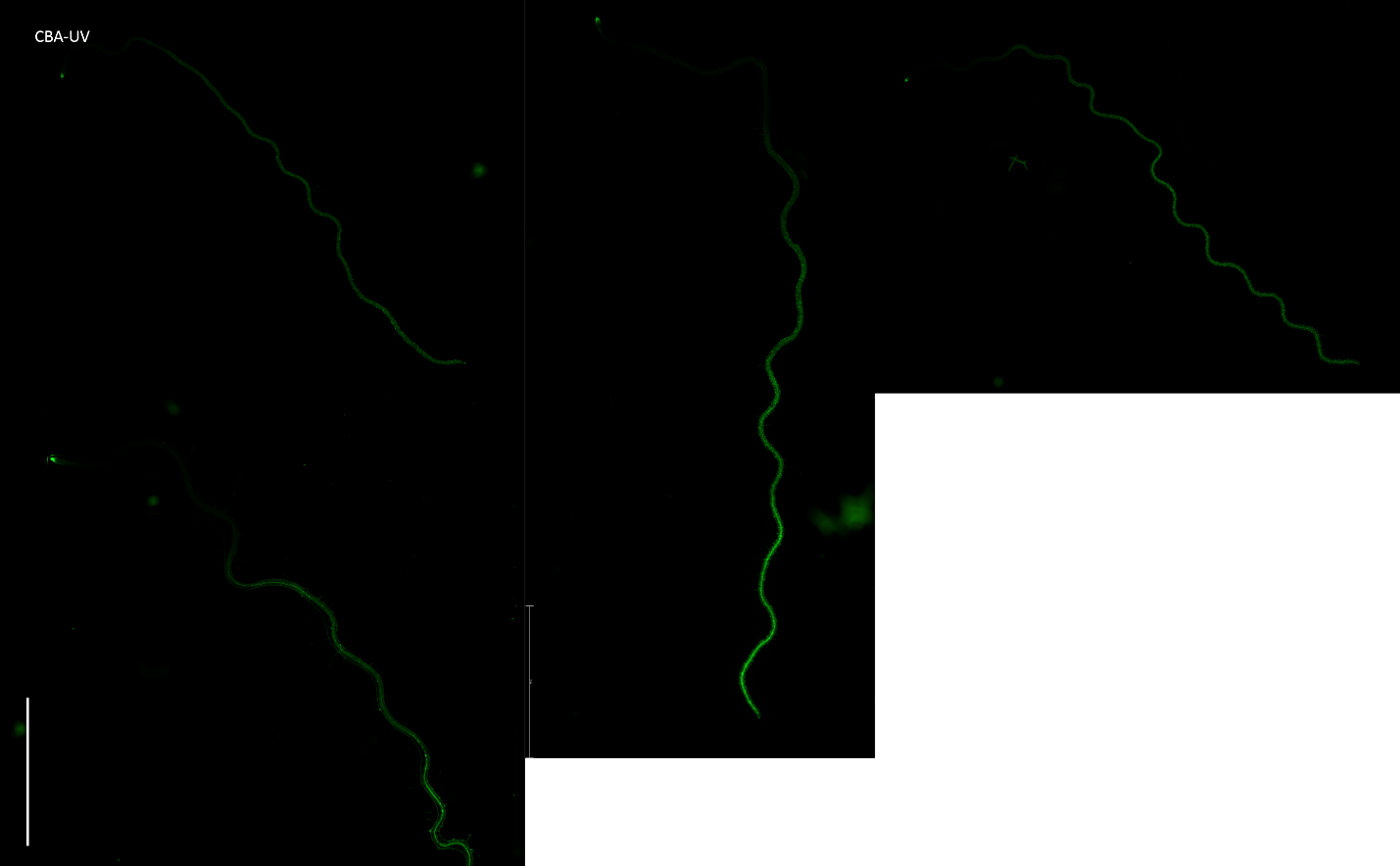


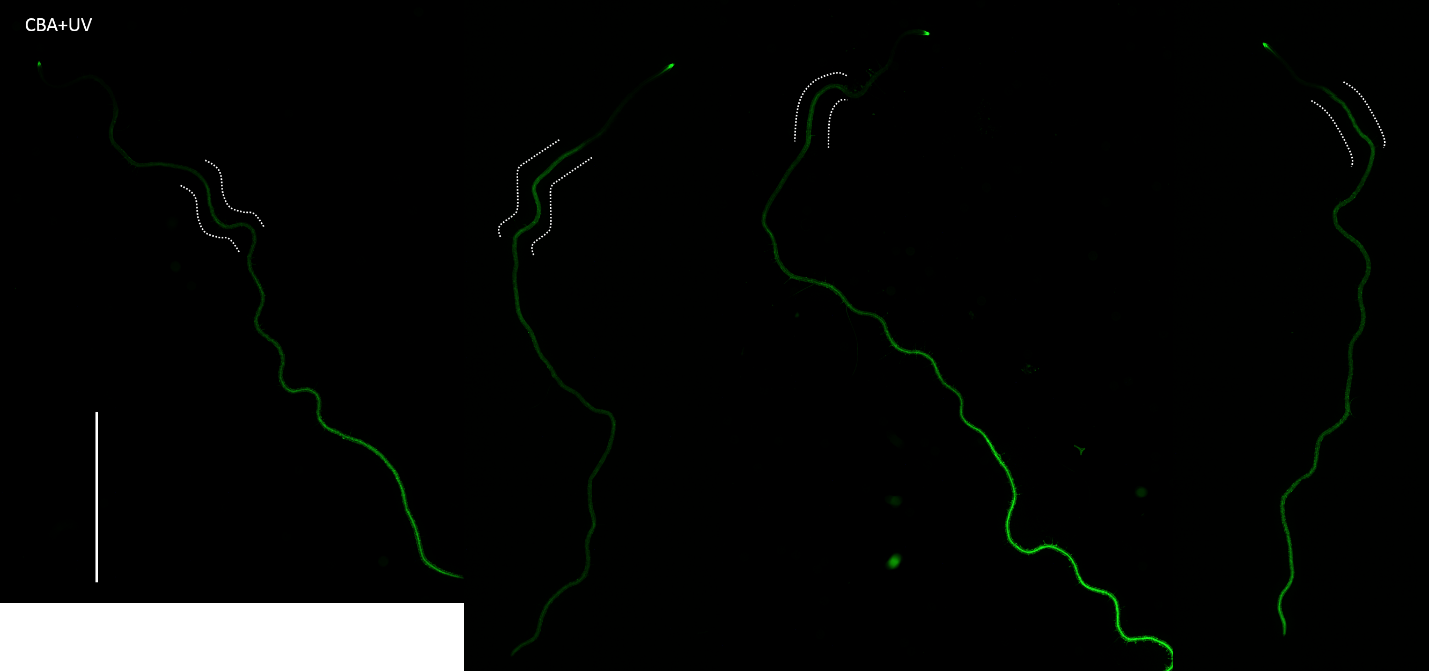


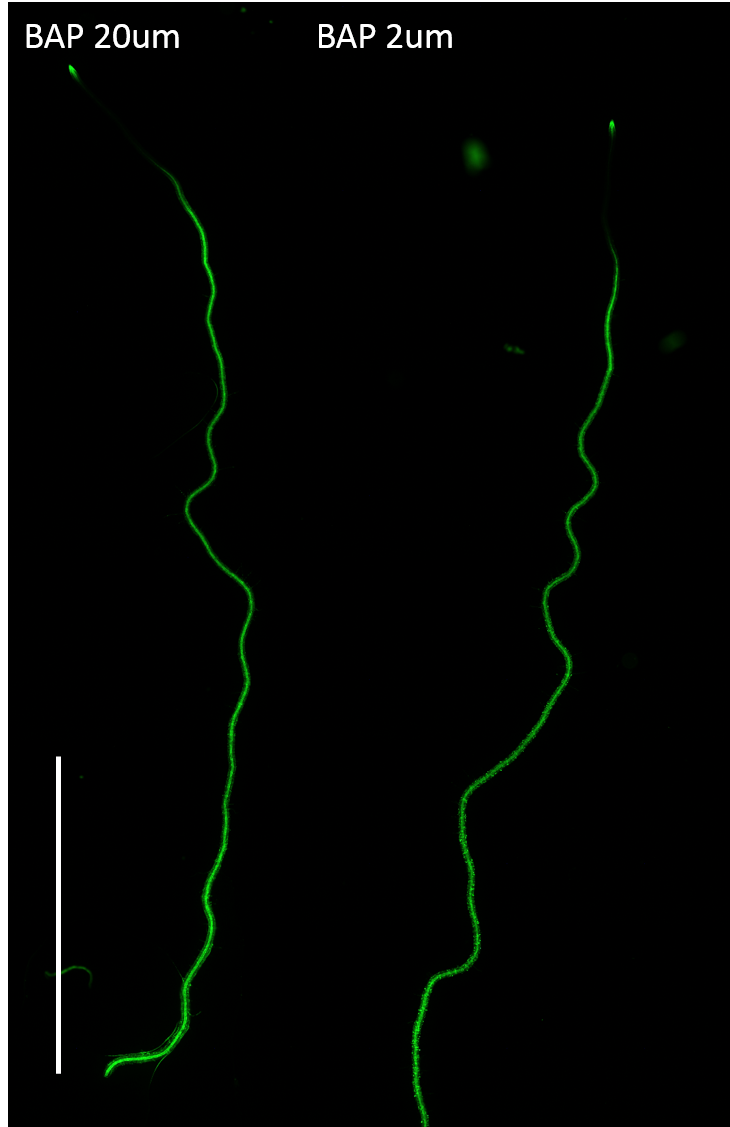

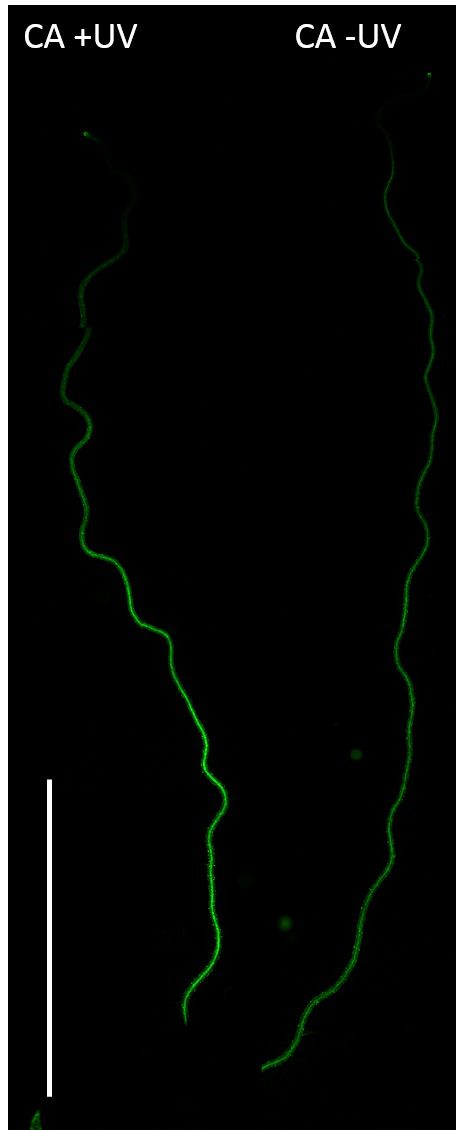


**Figure S14** Uncaging in small regions using a compound microscope. One-week-old *A. thaliana TCSn::GFP* seedlings were treated with a solution of 100 µM CBA, 100 µM caged adenine, or standard cytokinin (BAP). The solution was left to absorb for thirty minutes before being rinsed off. Respective seedlings were positioned under a Leica M205FA microscope and small sections were irradiated using the 10 × objective for ten minutes. After 16 hours the seedlings were mounted and imaged using fluorescence optics. Scale bars correspond to 2 mm. Dotted lines indicate the regions fluorescing, after being irradiated.


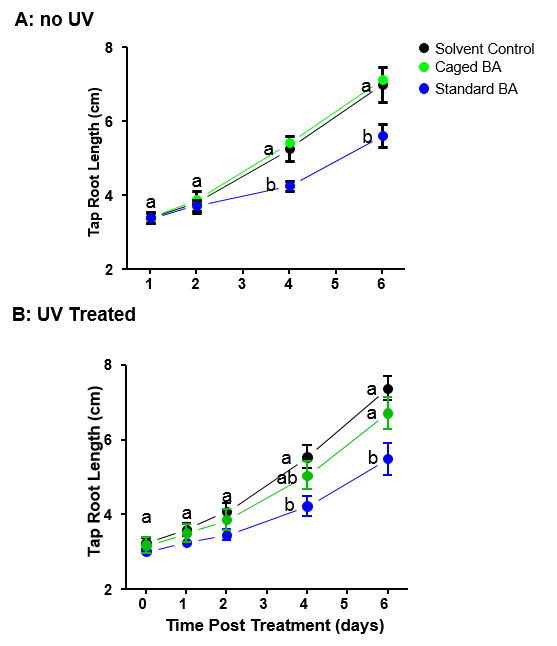


**Figure S15**: Uncaging CBA *in vivo* inhibits root growth. Wild-type *A. thaliana* seeds were germinated on standard half-strength MS media. At seven days post germination, 5 µL of either CBA (20 µM in 0.5% DMSO), a negative control (0.5% DMSO), or positive control (20 µM BA in 0.5% DMSO) were applied onto the root tip. The solution was left to absorb for 30 minutes, and was then rinsed with 5 µL of liquid half-strength MS media. Half of the seedlings of each treatment were then irradiated with 360 nm UV light from a Leica M205FA stereomicroscope for one minute. Root tip growth was then measured daily and analyzed using ImageJ. Results showed that without UV irradiation (**A**), no difference in root growth was observed between the CBA and solvent only treatments, however with UV irradiation (**B**), root growth was inhibited when treated with CBA, although not statistically significant. Bars indicate the means of eight seedlings. Error bars indicate standard error (SE). Treatments at each time point differing statistically by P<0.05 are indicated with different letters (two-way ANOVA).


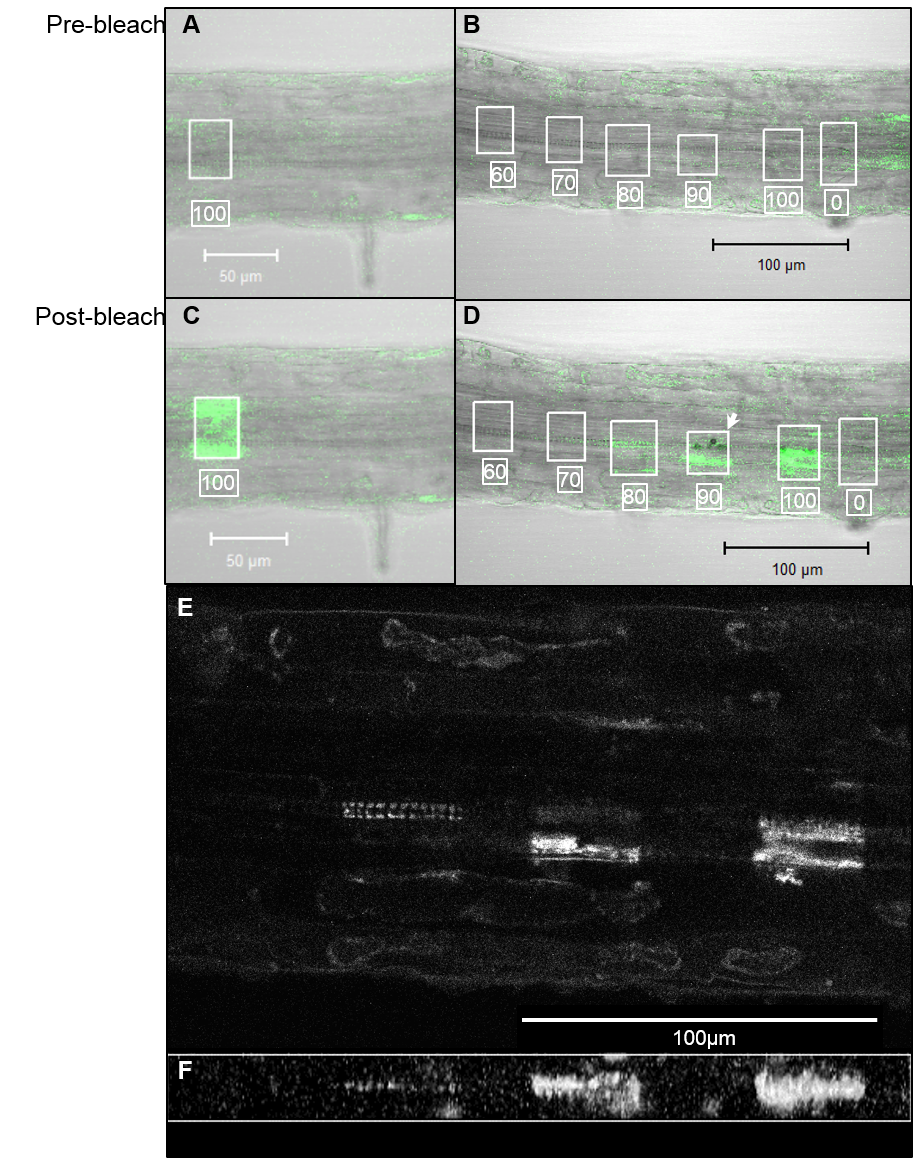


**Figure S16**: Detection of caged-fluorescein uncaging using a 720 nm (multiphoton) laser. Roots were incubated in caged fluorescein for 2 h, irradiated with the 720 nm MP laser in the regions shown (white boxes) then imaged with a 488 nm laser. **A & B**) Roots before uncaging. **C & D**) Roots immediately after uncaging, with green corresponding to fluorescein fluorescence. White numbers correspond to the laser power (%) used in each region. White arrow highlights possible photo-induced damage, seen as a darker region in the transmitted light background. **E & F**) Horizontal section from a single confocal plane (**E**) and corresponding vertical z reconstruction from confocal z-stack (**F**) of fluorescein emission post uncaging, showing vertical specificity of uncaging. Three distinct regions of fluorescence are visible, corresponding, from left to right, to the 80, 90 and 100 % laser intensity regions in **D**, showing strong fluorescence apparently localized to xylem elements.


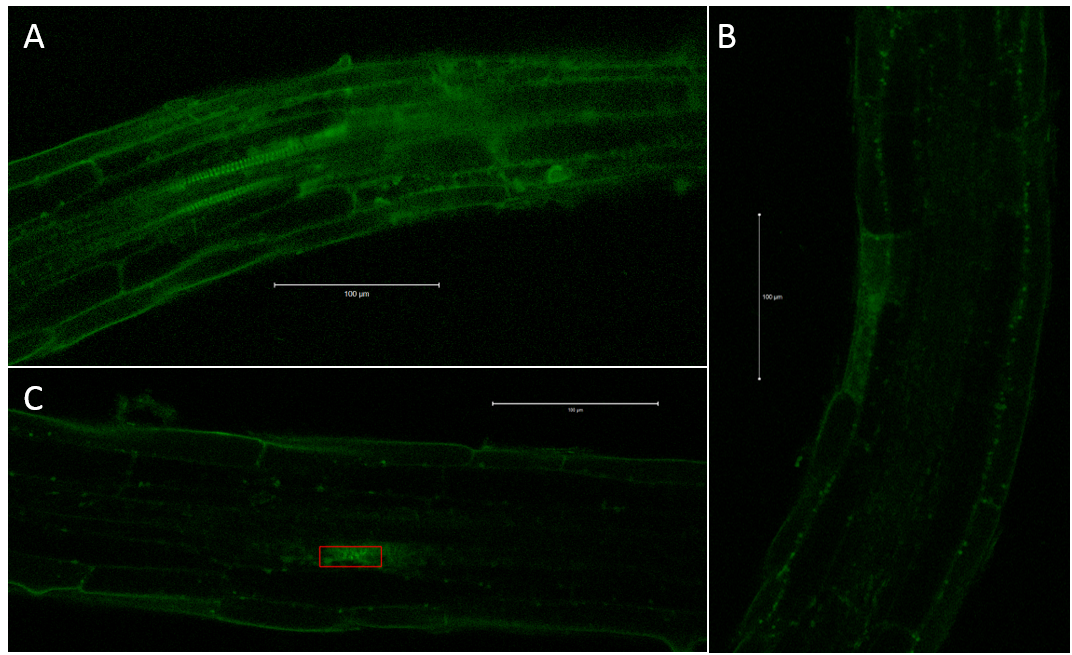


**Figure S17**: Further images of successful GFP response from tissue-specific uncaging of CBA. A) GFP response evident in vascular tissue. B) GFP response evident in epidermal cell. C) GFP response evident in endodermal cell.
